# Engaging the Guatemala Scientific Diaspora: The Power of Networking and Shared Learning

**DOI:** 10.3389/frma.2022.897670

**Published:** 2022-06-08

**Authors:** Kleinsy Bonilla, Claudia S. Romero-Oliva, Susana Arrechea, Nereyda Y. Ortiz Osejo, Sofia Mazariegos, Margarita Alonzo, Gabriela Orellana-Corrales, Andrea C. del Valle, Gabriela Montenegro-Bethancourt

**Affiliations:** ^1^Department of S&T Policies, Institute of Geosciences, University of Campinas, Campinas, Brazil; ^2^Organization of Women in Sciences for the Developing World Guatemala National Chapter OWSD Guatemala, Trieste, Italy; ^3^Centro de Estudios Atitlán, Universidad del Valle de Guatemala, Sololá, Guatemala; ^4^New Sun Road, P.B.C., Richmond, CA, United States; ^5^Department of Biosciences and Nutrition, Karolinska Institutet, Huddinge, Sweden; ^6^Wuqu' Kawoq | Maya Health Alliance - Tecpán, Chimaltenango, Guatemala

**Keywords:** science diasporas, S&T policy, S&T capacity building, Guatemala, diaspora knowledge networks, skilled migration, OWSD, brain drain-brain circulation

## Abstract

The underdevelopment of the higher education system in Guatemala and the fragility of its science and technology (S&T) contexts have compelled a significant number of talented Guatemalan scientists to be trained, educated, and employed abroad. The relocation of such skilled human power to different countries and regions has resulted in a growing Guatemalan Scientific Diaspora (GSD). Until recently, the emigration of scientists from the Global South to scientifically advanced countries in the North was studied as it negatively impacted the countries of origin. However, technological upgrades and globalization have progressively shifted the paradigm in which such scientific diasporas interact and connect, thus enabling them to influence their home countries positively. Due to the lack of knowledge-based evidence and functioning connecting platforms, the value and potential of the GSD in their involvement in proposing solutions to complex socio-economic, environmental, and other challenges faced by Guatemalan society remain unknown. Moreover, the lack of interaction of relevant stakeholders (S&T policy agents, international partners, higher education institutions and research centers, industry, and relevant not governmental organizations) represents a pervasive obstacle to the untapped impact of the GSD in the country. This study outlines the Guatemalan scientific diasporas' networking as a mechanism for building research excellence and intellectual capital. This force could respond to the need to strengthen the national science capacities and meet the demands for knowledge production and access to broader sectors of society. This research applied qualitative methodology that, through the conduction of focus group discussions and semi-structured interviews with members of the Guatemalan scientific community and relevant key stakeholders, delved into the existence and articulation of the GSD and potential stages for their engagement with their country of origin. Findings highlight the importance of digital and technological pathways that might leverage the GSD's knowledge and experience, channeling skills, and international connections for better interaction with the Guatemalan society. Furthermore, the discussion addresses how technology might turn brain drain into brain circulation, enabling the articulation of the GSD as a viable opportunity to generate collaboration between scientists abroad and local actors, ultimately impacting the building and development of Guatemalan science and national research capacities.

## Introduction

Guatemala is a developing country located in the Central American region. It is categorized as an upper-middle-income economy with a Gross National Income per capita of US$4,603 (World Bank, 2021). Although it is the largest economy in Central America, the limited scale of its geographic area (108,800 km^2^) and relatively small population reported at 15 million (Censo Población, 2018) pose challenges in terms of the development and strength of the national scientific and research context. Despite its growing economy, Guatemala is a country with poor development indicators with limited local economic opportunities; thus, international migration is an option that -at the individual and household level- is perceived as an opportunity to improve the home economy (Saenz de Tejada, 2009; Canales et al., 2019). Moreover, during the internal civil conflict (1966–1996), there were massive emigration waves of Guatemalans, especially from the most affected areas (i.e., the highlands and rural areas) to Mexico and the United States of America (ICEFI, 2021). This international migration included scientists, lecturers, and intellectuals subjected to political persecution. In other words, a combination of lack of work training and job opportunities, and a violent and hostile local context, have produced a constant exodus of Guatemalans (both low and highly skilled human resources). Canales et al. (2019) have estimated the loss of nearly 30.0% of highly skilled Guatemalan migrants, doubling the 13.5% estimated in Commander et al. (2004). Admittedly, the deepening of globalization, the emergence of a digital era, and advances in technology have resulted in the possibility of turning such loss into opportunities, particularly considering frequent and multiple networking collaborations.

### Migration of Highly Skilled Human Capital

According to the Organization for Economic Cooperation and Development, OECD (2016), there is increasing global competition for talent, reflecting that highly educated individuals participate in mobility schemes and are prone to emigration. It is possible to identify strategic international open networks that attract foreign talents to reduce skill gaps, particularly for smaller or lower-income countries. Scientific and research migrations have become more complex in the global landscape, making high- and low-income countries pay more attention to policies that offer more attractive conditions to support inward, outward, and return migrations. However, the “brain drain” paradigm continues to be dominant in the literature when addressing the mobility of skilled human capital from the Global South to the North (Brown, 2002; Davis and Weinstein, 2002; Commander et al., 2004). Brain drain has consolidated as a critical issue for developing countries that struggle to position themselves in the global market. According to Brown (2002), globalization changed the global economy from more labor-intensive to more knowledge and technology-intensive industries.

High development indicators, along with the country's size and proximity, are among the main determinants of emigration, for which small and developing countries observe the highest rates of skilled emigration (Docquier et al., 2007; Docquier and Rapoport, 2012). The primary motivations of scientists and academics to migrate include having access to higher scientific and technological infrastructure and career opportunities and, to a lesser degree, a higher human development index (Siekierski et al., 2018). In the past, the relocation of such valuable human power from the Global South to scientifically advanced countries was considered to negatively impact the countries of origin, as reflected by it being termed “brain drain”: a loss in highly skilled human capital. Nevertheless, technological evolution has changed the paradigm in which such scientific diasporas interact and connect, thus enabling a positive influence on the development of the country of origin. Indeed, the current trend regarding these migration patterns is to conceptualize them as “brain circulation,” or a “triangular flow of human talent,” by which high skilled migration is posited as benefiting both the emigrating and immigrating country, rather than benefiting only one at the expense of the other (Tung, 2008). In an interesting analysis from Defoort (2008), it was concluded that brain drain has increased through time (mostly from 1990 to 2000). Long-term migration of highly skilled workers 25 years of age and older from the Central American region into the six main receiving countries (United States, Canada, Australia, United Kingdom, Germany, and France) between 1975 and 2000 was the third highest globally and second in Latin America, just behind the Caribbean. Since 1975 the main destination of highly skilled emigration from Central America has been the United States of America, with 98.8% of total skilled emigration rates. Moreover, in the year 2000, four out of the seven countries in Central America rank as 9th (El Salvador 30.4%), 10th (Nicaragua 28.8%), 17th (Honduras 23.9%), and 18th (Guatemala 23.5%) in the countries with a population size between 2.5 and 20 million inhabitants with the highest brain drain. The analysis of Lozano and Gandini (2011) portrays these differences by analyzing data from 1990 to 2007 from these regions. The difference strives in how skilled migration in less populated or smaller countries such as Haiti, Dominican Republic (33.0%), Belize, Nicaragua, and Honduras (25.0%) appear higher than in highly populated and bigger (10.0% or less) countries like Brazil, Venezuela, Colombia, and Peru, just to name some. In specific reference to the Central American and Caribbean Region, including the case of Mexico, there is evidence that the highly skilled migration target is mainly the United States of America, Canada and Mexico, with Mexico as the country with the highest skilled migration rate of all Latin-American countries (increasing emigration rates 3.7 times from 1990 to 2007, 207%) (OIM, 2019). In terms of the educational level of the highly skilled migrants, data surprisingly showed that more skilled migrants born outside the United States of America have a doctorate compared to US citizens. This was not the case for those holding a bachelor's, where the opposite trend was evident. Furthermore, skilled migrants having a gainful job based on their qualifications in the United States was the following, according to their academic level: with a bachelor's degree 68.2% (at a higher disadvantage), master's degree 20.4% and a doctorate 11.4%. Likewise, a so-called “brain waste” refers to the underemployment of skilled migrants who work in occupations whose qualification requirements are below their education levels (Özden, 2005). In this sense, in all regions of the world, unemployment is higher among skilled immigrants as citizens [4.0 and 3.2% for those originally from Africa and Latin America (4.2% for Guatemala), respectively] (Lozano and Gandini, 2011). The literature describes the interaction between host countries and highly skilled migrants as a “massive phenomenon” in terms of the economic and human resources potential for both the target and origin countries. It is such that more countries have identified their potential and is now part of the strategic plan proposed by the OECD in their 2035 future assessment in skilled migration (OECD, 2020). Such tendencies and views are still not that positive for the Latin American and the Caribbean (LAC) region. For instance, demographic data retrieved from the United States of America (the main destination for highly skilled migrants in the LAC region) until 2011 indicates that the labor increment of highly skilled migrants is still lower than that of citizens with a higher tendency toward increased educational level. It is necessary to highlight that these data might not explicitly represent the perspective and view of all countries hosting the Guatemalan Scientific Diaspora (GSD). Thus, the presented outcome could explain a gap between national policies, perception, international relations, and the labor market.

### Approaches to the Term Diaspora in the Context of Science

According to the International Organization for Migration (IOM), a diaspora has been defined as a general concept referring to ethnic persons or populations, individuals, and members of networks and organized associations who left their homelands of origin and maintained a connection with their countries (OIM, 2021). IOM emphasizes the transnational dimension of diasporas, the relation between their country of origin and destination, rather than the historical connotation. On the other hand, scientific diasporas (SD) have been defined as “self-organized communities of expatriate scientists and engineers working to develop their home country or region, mainly in science, technology, and education” (Barre et al., 2003, p. 15). For Tejada (2012), scientific diasporas are made up of emigrated scientists, and skilled professionals who have gained recognition as promoters of research and communicators of knowledge contributing to scientific, technological, and socio-economic development in their home countries. During the last decades, various countries have been developing policies to counteract the brain drain by attracting highly skilled people to their country of origin through incentive schemes without success. For Tejada et al. (2013), large-scale emigration by scientists and qualified professionals from developing and transition countries in search of better opportunities and career prospects in high-income industrialized countries, commonly known as “brain drain,” is a significant concern for their respective home countries. Nonetheless, the emigrated human capital can also bridge the home and host countries, promoting the transfer of ideas, skills, and knowledge. Burns (2013) considers science diasporas as engines of innovation, as in the United States of America, a quarter of foreign-born workers with college degrees work as scientists or engineers. In Silicon Valley, California, 44.0% of these engineering and technology ventures were founded by at least one immigrant (Burns, 2013). This is consistent with Meyer (2007) and Tejada (2012), who identify the power of the scientific diaspora as a driver for development and improvement. The concept of scientific diasporas started to reference networks or organizations of emigrated scientists and engineers from developing countries living in industrialized countries (Barre et al., 2003). Such networks or organizations were thought to work together to “transfer knowledge to their countries of origin through diverse forms of cooperation from a distance” (Tejada, 2012, p. 61). Another relevant concept is diaspora knowledge networks (DKN). According to Meyer (2007), DKN provides new policy options in innovation, science and technology, migration and development, and international cooperation. Brown (2002) argues that scientists leave their home countries to study or work in an industrialized country to acquire knowledge and expertise they might not have gained if they remained at home. They also establish knowledge and information networks in the host country. For Meyer and Brown (1999), the rise of DNK during the 90s portrayed a potential resource for practical cooperation between developing and highly industrialized countries. For the last two decades, the conception about the migration of skills evolved from brain drain to brain gain (The National Science Technology Portal of the Republic of Belarus, 2020). Some countries, as in the case of Belarus, promote policies to discourage university graduate students from migrating, especially those who studied in a public university, as a result of certain repay dispositions to the Education Code in 2011, in which “graduates from state universities and specialized secondary educational institutions where the tuition for their study was paid by the state have to work for an employer assigned by the state for two years following graduation” (CASE, Kazmierkiewicz and Kulesa et al., 2021, p. 23). The stance of populist governments in the Global North in recent years has created a new paradigm in skilled migration. This new paradigm is called “brain rejection,” which refers to the rejection of highly skilled migrants in traditional destination countries such as the United States or the United Kingdom. These countries have questioned the benefits of the brain gain strategy for developing the recipient country. The reasoning behind “brain rejection” in populist narratives is protectionism of culture and the economy, especially when it comes to native workers (Tigau, 2020). Most of the literature has focused on the voluntary and economic migration of highly skilled migrants. However, some professionals flee their home countries due to conflicts and wars (Tigau, 2019). Refugees are not always poor and uneducated, but a good part of them are well-educated and successful people who migrate forcibly due to war circumstances (Tigau, 2019). A survey of 305 Syrian refugees in the UK, the Netherlands, and Austria shows that 38.0% have a university degree (Betts et al., 2017). Bang and Mitra (2013) find that ethnic civil wars significantly impact the magnitude of skilled migration, while non-ethnic wars do not have a strong and significant effect. Ethnic wars increase the number of high-skilled migrants by 5.0–8.0%, and each additional year of war increases the share of high-skilled migrants by 0.4–0.7% (Bang and Mitra, 2013). One of the main challenges of highly skilled refugees is finding a job due to lack of institutional support, language barrier, and assimilation obstacles (Betts et al., 2017). However, Tigau (2019) suggests that changing the perspective on refugees from burden to “boon” would allow professional refugees to be included in destination countries' brain gain strategy. In the same way, highly skilled refugees can be seen as potential positive contributors to conflict mitigation, de-escalation, and even resolution and post-conflict reconstruction in their home countries (Bercovitch, 2007; Koser, 2007; Bang and Mitra, 2013).

### Experiences in the Scientific Diaspora in Latin America

In the case of Latin America, some countries have been adapting their policies regarding their diaspora to maximize the potential contribution to local development. In 2007, 94.0% of governments held policies and programs for their diaspora residing abroad, mainly in the United States of America (OIM, 2021). In the 1980s and 1990s, Mexican science experienced a period of expansion with the incorporation of researchers trained abroad, establishing the National System of Researchers to stop the flow of scientists abroad (Marmolejo-Leyva et al., 2015). The case of the Mexican Scientific Diaspora suggests that the mobility of Mexican researchers had a substantial impact on their production and the extent of their scientific collaboration. Some of them maintain their research engagement when they return. Marmolejo-Leyva et al. (2015) also found significant differences among areas of knowledge, where the most productive researchers are those in biological sciences, physics, and engineering. Indeed, the case of Mexico offers an interesting example in Latin America of the contribution of the scientific and technological development of their home country (Rivero and Trejo-Peña, 2020). A recent study by Marmolejo-Leyva et al. (2015) identified that a high number of scientific productions in collaboration with countries from the Global North, mainly the United States of America and the United Kingdom, was produced between 2003 and 2009. This occurred after high global migration rates register in between 1990 and 2007: the “stock” of highly skilled professionals with origins in Latin American and Caribbean countries increased 155.0%, followed just by Africa and Asia with 152.0 and 145.0%, respectively (Tejada and Bolay, 2005; OIM, 2019). The key to the collaboration of the Mexican Scientific Diaspora with their country of origin relies on the ties it still maintains with Mexican institutions. These have resulted from a few governmental return and repatriation policies, such as the National System of Researchers (SNI-for its acronym in Spanish) from the National Council for Science and Technology (CONACyT-for its acronym in Spanish) created in 1984 by a presidential decree recognizing the scientific career of researchers and providing economic stimuli. In 2003, the Institute of Mexicans living abroad was created, and in 2005, the Chamber of Deputies passed a law to support former Mexican migrant workers. Finally, some independent initiatives from non-governmental institutions have arisen, like the Red Global MX, which bridges relationships between highly skilled Mexicans and local institutions. There is still much to do regarding return policies, as many interviewed scientists highlighted that no real investment in science and technology exists; only superficial incentives are provided to scientists from the SNI system. Moreover, incipient repatriation programs are offered to highly skilled workers (Rivero and Trejo-Peña, 2020). Also, in Colombia, the Colombian Ministry of Science, Technology and Innovation (Miniciencias for its acronym in Spanish) promotes access to master's and doctoral programs abroad, nevertheless repatriation or engagement actions subsequent to their completion is very weak (Echeverría-King, 2021). In the case of Central America, diaspora organizations are often involved in activities focused on their country of origin. They range from small donations to investments and infrastructure projects and in the case of Guatemala 31%, organizations related to their diaspora carry out food support actions in favor of their communities of origin (OIM, 2021). The National Council for Attention to Migrants (CONAMIGUA) is among the organizations that provide attention, engagement, and participation with the diaspora, mapped by the IOM, which has a registry of irregular migrants that is not publicly accessible and does not include the GSD.

Notwithstanding, research on how the needs and perceptions of the scientific community (i.e., diasporas, returned scientists, and local peers) dialogue with stakeholders back in their home countries is lacking. To plan for any future policy engagement, this research aimed to outline the potential of the GSD network as a mechanism for building research excellence and intellectual capital while also delving into the existence and accumulation of the GSD and stages for their engagement with their country of origin. Furthermore, it aimed to address how technology might turn “brain drain” into “brain circulation” and enable the articulation of the GSD as a viable opportunity to generate collaboration between scientists abroad and local actors, ultimately impacting the building and development of the Guatemalan scientific capacities. This research sheds light on the current state of the mapping, characterization, and identification of the GSD for their engagement and interactions with their peers, relevant stakeholders, and broader sectors of the society back in their country of origin.

## Methodology

The research was conducted using a qualitative methodology. Data was collected from two groups of participants: (i) The Scientific Community with emphasis on members of the GSD, and (ii) the perspective of a comprehensive group of Stakeholders with relevance to the GSD. To select the first group, a strict set of criteria was designed to guarantee a diversity of views and perspectives (Table 1).

**Table 1:** Criteria—selection of key respondents of semi-structured interviews scientific community.

**Criteria**	**Description**	**Operationalization**
Experience	Experience in community building or participation, networking, groups of scientists	Reporting experience in building and/or participating
Trajectory	Procure diversity in the representation of career development stages of the interviewees (early, mid-established career)	Years since completion of graduate studies. Early <10 years, Mid +10 years but no management positions or group coordinators. Established +15 years in addition to management or research group coordination positions
Field of Expertise	Diverse fields of knowledge (i.e., natural sciences, health, earth science, social sciences, physics, engineering sciences)	All fields of knowledge were considered, including social, natural, and engineering sciences
Destination Diversity	Covering a wide range of geographic locations for destination	Including as many geographic destinations as possible region/country, i.e., North America, Europe, Asia, Latin America
Gender Balanced	Balanced participation of women and men	Gender equality in the participation

In the case of the second group, a categorization of six perspectives was followed to include an integral approach (Table 2). The presented data was collected between September 2021 and February 2022.

**Table 2:** Perspectives and stakeholders relevant to the GSD.

**Perspective**	**Profile of the stakeholder**
Science technology and innovation policy	Institution/Organization relevant to the Science, Technology, and Innovation Policies in Guatemala, e.g. The Guatemala National Secretariat of Science and Technology SENACYT, the Secretariat for Planning and Programming of the Presidency SEGEPLAN, the Commissions of Science, Technology and Education in the National Congress, Association of the Agricultural Chemical Guild (Agrequima)
Foreign policy	Institution/Organization relevant to Guatemala's foreign policy. e.g., Ministry of Foreign Affairs, Central American Parliament, Commission on Migrants and Commission of International Affairs of the National Congress
International partner	Institution/Organization engaged in science and technology international cooperation with Guatemala, e.g., UNESCO, Foreign Missions accredited in Guatemala, the Central American Integration System SICA
Higher education/research institutions	Universities with full-time research positions/Public or Private Research Center, San Carlos of Guatemala University, Del Valle de Guatemala University, Rafael Landivar University, Mariano Galvez University, the Central American Council of Higher Education CSUCA
Industry/private sector	Organization/Firm from the private sector engaged in Research and Development Activities, i.e., Cementos Progreso (Cetec, research institute), Agexport (Network I+D+i), Cámara de la Industria (Industry Chamber), AGEXPORT, Cámara del Agro (Agribusiness Chamber) and CAB-Corpo, CNE GT (Consejo Nacional Empresarial)
Social/ civic organizations	Organizations from the organized Civil Society. e.g., Institute for the Development of Higher Education (INDESGUA), Fundación Desarrolla Guatemala to the acronym (FUNDEGUA), Demos2025, the Luis Vohn Ann Foundation

As for the methods for data collection, participants were offered two alternatives (considering their time availability): (i) Semi-structured interviews and (ii) Participation in Focus Group Discussions. Tables 3, 4 present the profiles of the participant Scientists and the Stakeholders, respectively.

**Table 3:** Participants from the Scientific Community: method, demographic distribution and fulfillment of the selection criteria.

**Type of activity and demographic distribution**	**Participatory method employed**	**Participant selection criteria**
*Focus Group Discussion* Total 18 **Gender**: Female 10; Male 8; other 0 **Field of expertise**: Biomedical sciences 2; Business and Innovation 1; Educational Sciences 1; Engineering and Computational Sciences 3; Environmental Sciences 7; Physics 1; Social and Political Sciences 3 **Geographical location**: Asia 2; Oceania (incl. Australia) 0; Central America 3; Europe 6; North America 4; South America 3 *Semi-structured interviews* Total 19 **Gender**: Female 12; Male 7; other 0 **Field of expertise**: Biomedical sciences 5; Business and Innovation 1; Educational Sciences 1; Engineering and Computational Sciences 5; Environmental Sciences 4; Physics 1; Social and Political Sciences 2 **Geographical location**: Asia 3; Oceania (incl. Australia) 1; Central America 1; Europe 7; North America 7; South America 0	*Online Focus Group Discussion* 6 focus groups with different numbers of participants fitting the criteria selection (Group [G] 1:5, G2:4, G3:2, G4:2, G5:3, G6:2). Average duration 60 min *Semi-structured interview* One-to-one interviews with an average duration of 45 min	*Trajectory* 1. early career 2. mid-career and 3. established career

**Table 4:** Participants from the stakeholders: method, demographic distribution and fulfillment of the selection criteria.

**Type of activity and demographic distribution**	**Participatory method employed**	**Participant selection criteria: perspective**
*Focus Group Discussion* Total 26 (Female 11 Male 15) Gender: Female 12; Male 13; other 0 *Semi-structured interviews* Total 11 (Male 6 Female 5) Female 5; Male 7; other 0	*Online Focus Group Discussion* 6 focus groups with different numbers of participants fitting the criteria selection (G 1:4, G2:7, G3:3, G4:6, G5:3, G6:3). Average duration 60 min *Semi-structured interview* One-to-one interviews with an average duration of 45 min	*Perspective* 1. Science and technology policy 2. Foreign policy 3. International partner 4. Higher education/Research institutions 5. Industry/Private sector 6. Social/Civic organizations

To fulfill the general objective of this study, literature review and desk research was carried out in complement to the collection of primary data. All interviews and focus group discussions recorded used different digital platforms and software (i.e., Google-meets, Zoom, and WhatsApp). Semi-structured interviews averaged 45 min, whereas focus group discussions averaged 60 min. A total of 30 h of audio-visual material were recorded. All materials were transcribed into text files (by listening and directly transcribing), codified, and analyzed to determine patterns, trends, shared content, and contrasting views. Table 5 summarizes the criteria and sub-criteria used to analyze the data.

**Table 5:** Topic criteria and sub-criteria for data analysis.

**Topic**	**Criteria**	**Sub-criteria**	**Terminology coded (key words) in each sub-criterion**	
Identification	Current situation		Not aware of databases/mapping, *Converciencia* (yearly scientific event in Guatemala) Red CTI (International Network of Guatemalan Scientists)	OWSD (Organization of Women in Science in Developing Countries) SENACYT (National Directorate of Science and Technology), Fulbright (United States of America scholarship program)
	Limitations and critique		Lack of: Interests/attention Linkage	Maturity Policies Data
	Proposals and technological potential		Related databases Social Networks to map (Facebook, LinkedIn, Research Gate)	Data mining
Connection	Current situation		Lack of experience No collaboration Mentoring activities	Scientific dissemination Collaboration in projects and scientific publications
	Limitations and critique		Lack of: Connection/linkage Interinstitutional coordination	Communication Normative Transparency
	Proposals and technological potential		Related social networks (Facebook, LinkedIn, ResearchGate) Communication technologies (Whatsapp, Zoom, Meet, BlueJeans, Slack)	Articles and publication platforms (Scopus, Academia) Database of scholarship holders Video platforms (TedEx, YouTube)
Engagement	Current situation	Diplomacy	Lack of programs from the Ministry of Foreign Affairs Embassies or Missions	Fulbright programs
		Governmental institutions	Strengthen programs and networks	Lack of political will
		Scientific communities, OWSD, Academia, Civil society	OWSD activities *Converciencia* activities Scholarship holder networks	INDESGUA (Higher education development institute, in Spanish) database
		Universities and Academies	Projects Exchange	Observatory Policies
		Industry	Industry Chamber Partnership	*Guatemaltecos ilustres* (Prestigious prize for recognized Guatemalan professionals)
	Limitations and critique	Diplomacy	Lack of: Incentives/rewards Coordination	Language Interest
		Governmental institutions	Lack of: Organization	Investment Policies
		Scientific communities, OWSD, Academia, Civil society	Lack of working tables with more sectors Lack of Involvement	
		Universities and Academies	Communication Infrastructure	Inter-institutional collaboration Trust
		Industry	Communication	
	Proposals and technological potential	Diplomacy	Communication	Will
		Governmental institutions	Communication	Identified needs
		Scientific communities, OWSD Organization of Women in Science for the Developing World, Academia, Civil society	Integration strategy	Dissemination
		Universities and Academies	Dissemination	
		Industry	Communication	

The study was reviewed and approved by the Ethics Committee at the University of Technology of El Salvador (UTEC). All participants were asked to sign an electronic informed consent form before participating in the study. Identities of participants are not identifiable nor traceable; all transcripts were encoded, and recorded material was shared only among members of the research team.

## Results

### Mapping of the Guatemalan Scientific Diasporas

**Figure 1:**
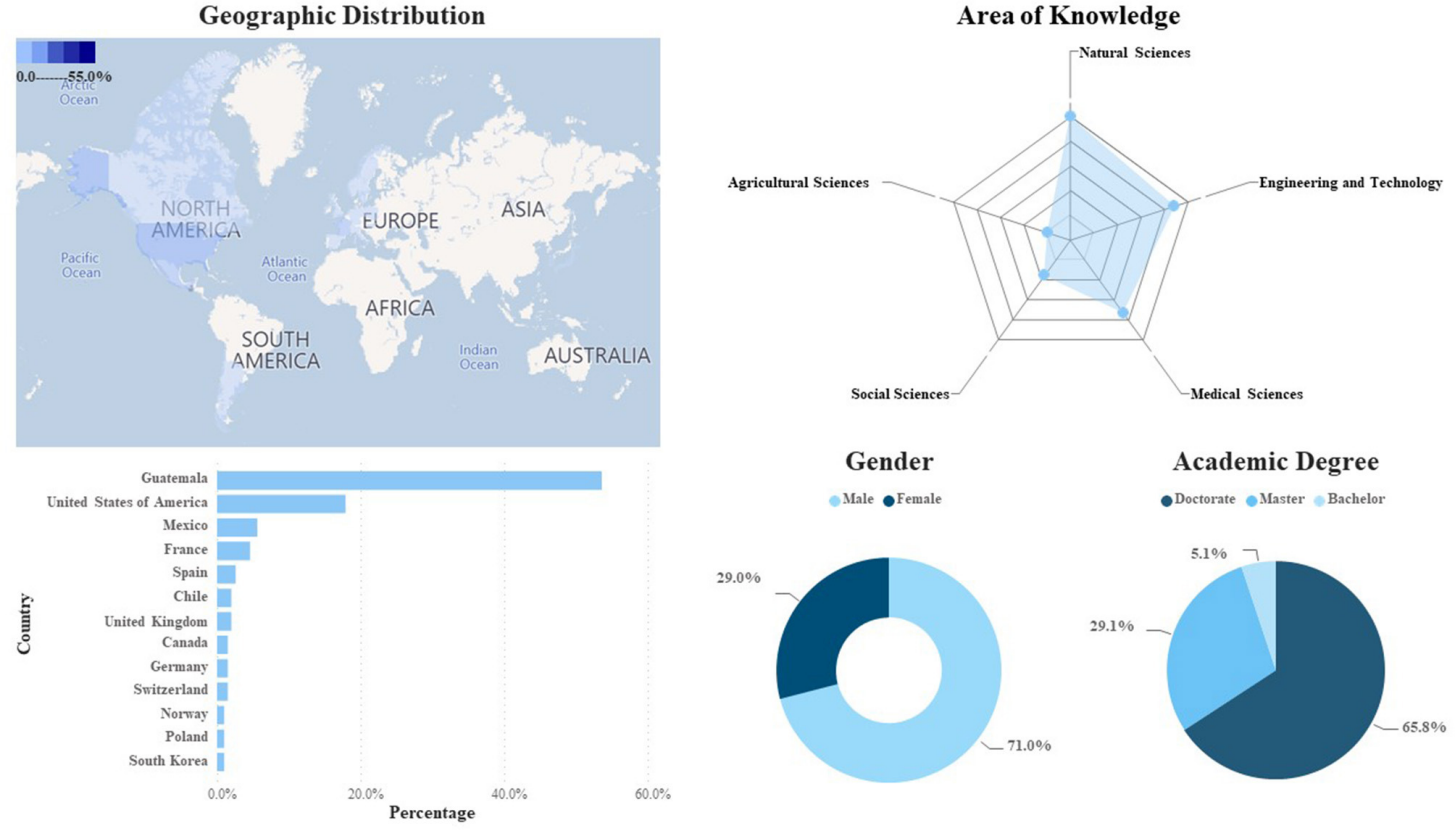
Mapping of the GSD—based in two networks RedCTI/OWSD Guatemala.

While mapping the GSD, two databases were identified and obtained. They were complemented with scientists of Guatemalan origin from different career trajectories both residing in Guatemala and abroad. Such information was publicly available. The two databases are from registered members of two networks of Guatemalan scientists: First, the International Network of Science, Technology and Innovation of Guatemala and second, the Organization of Women in Science from the Developing World-Guatemalan Chapter (OWSD-Guatemala). Demographic information gathered from these databases included: (a) gender (female, male), (b) last completed academic degree, (c) field of knowledge/expertise (i.e., natural sciences, social sciences, computer sciences, health, education, etc.), and (d) geographical location of their place of residence. Descriptive statistics were conducted to determine percentages, means, and standard deviations from the gathered data. The information available from the two databases summed up 631 scientists, out of which 78.0% (*n* = 491) are female. Seventy percent (*n* = 441) reported as country of residence Guatemala (female *n* = 392 and male *n* = 140) living in 33 countries, including Guatemala. In relation to their country of residency and work of the GSD, both North America (United States of America *n* = 58, Mexico *n* = 20 and Canada *n* = 3) and Europe (France and Germany both *n* = 14, Spain *n* = 13, United Kingdom *n* = 9, among the main) followed by Asia 1.1% (*n* = 7), Oceania 0.5% (incl. Australia *n* = 4) and no reports from countries in the African continent (Figure 1).

#### International Network of Science, Technology and Innovation of Guatemala—RedCTI

In 2005, the National Council of Science and Technology (CONCYT) brought together a group of Guatemalan scientists who worked on research activities and had their place of residence outside of the Guatemalan territory. This action aimed at “strengthening the science and technology capacities in Guatemala to ultimately propose solutions to the problems in the Guatemalan society”^1^. The meeting was called *Converciencia* and resulted in the foundation of the International Network of Science, Technology and Innovation of Guatemala (*RedCTI*) by signing a Constitutive Act during the event's closing ceremony. The *RedCTI* Network has been in operation since its creation (over 15 years) with the support of the National Secretariat of Science and Technology (SENACYT). The SENACYT is the coordinating public institution. It is responsible for supporting and implementing the decisions that emanate from the CONCYT (for its acronym in Spanish)^2^.

**Figure 2:**
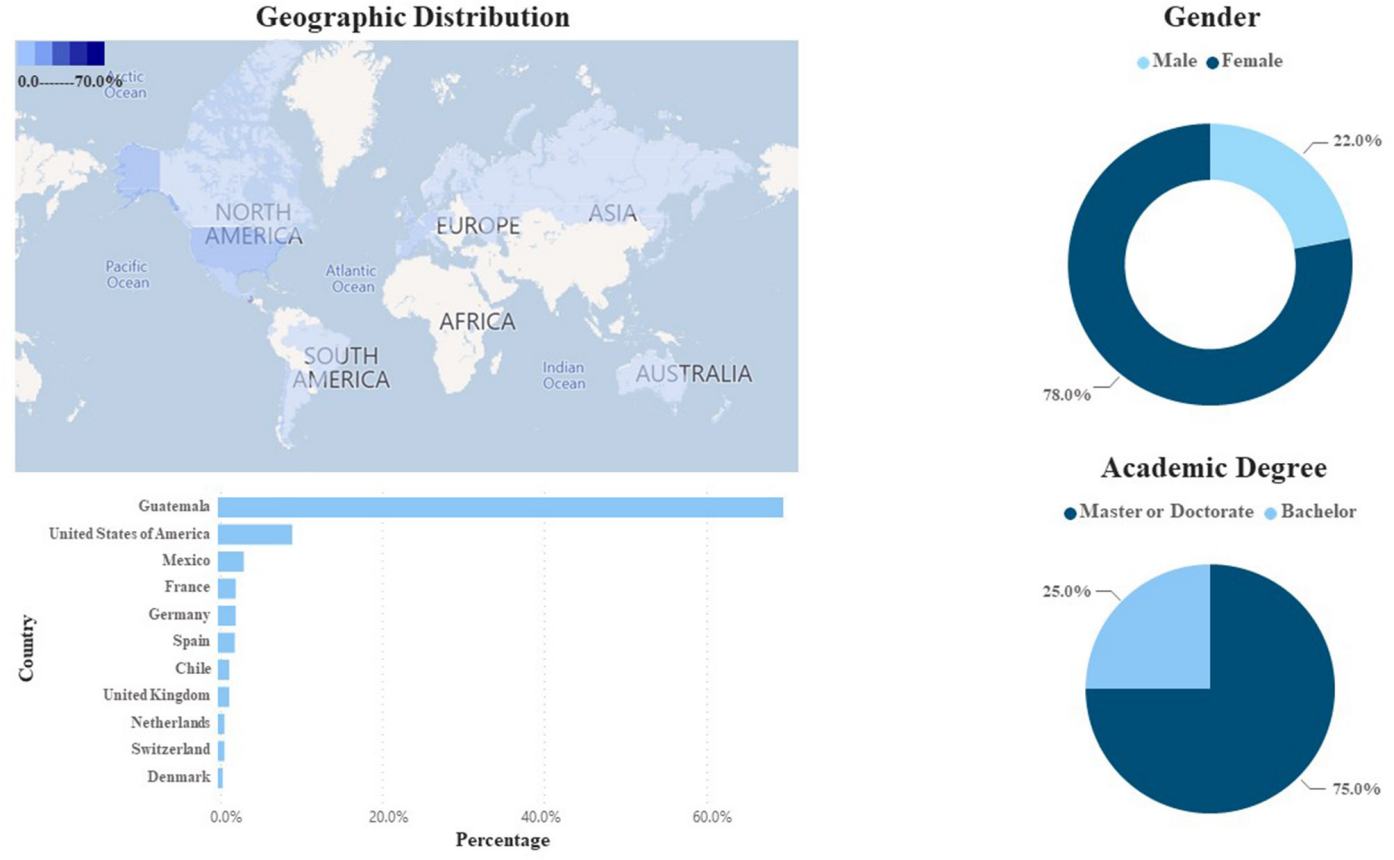
Geographical, area of knowledge, gender, and academic degree distribution of the scientists of the international network of Guatemalan scientists RedCTI (February 2022).

The *RedCTI* was created to contribute to the preparation and implementation of scientific-technological development plans through science, technology, and innovation and propose viable alternative solutions to improve the population's quality of life. Moreover, one of its main goals is to link Guatemalan scientists working outside with those inside the country. As of February 2022, this network registered a total of 196 members, according to the database provided by SENACYT. The directory allows mapping the GSD according to their gender, field of knowledge, and country of residence. Applying a binary gender filter (female/male) in this network, 71.4% (*n* = 140) are male and 28.6% female (*n* = 56). According to their field of knowledge professionals reported in this network includes 23.5 (*n* = 46) in the Medical Sciences 31.6% (*n* = 62) in the Natural Sciences, 27.6% (*n* = 54) in Engineering and Technology, 11.2% (*n* = 22) in Social Sciences, and 6.1 % (*n* = 12) in Agricultural Sciences. In terms of the level of education among its members, 5.1% (*n* = 10) reported having completed a bachelor's degree (*licentiate*), 29.1% (*n* = 57) master's degree, while 65.8% (*n* = 129) hold doctoral degrees (Ph.D.). Interesting data refers to the reported country of residence 53.6% (*n* = 105) reported their country of residence as Guatemala, while 46.2% (*n* = 91) reside abroad. In this aspect, a more comprehensive concentration of GSD is found in the United States of America with 17.9% (*n* = 35), followed by Mexico at 5.6% (*n* = 11) and 4.6% in France (*n* = 9). Other destinations with less presence include Argentina, Puerto Rico, Hungary, Italy, Netherlands, Sweden, Japan, and Taiwan (Figure 2).

#### The Organization of Women in Science for the Developing World Guatemala National Chapter

**Figure 3:**
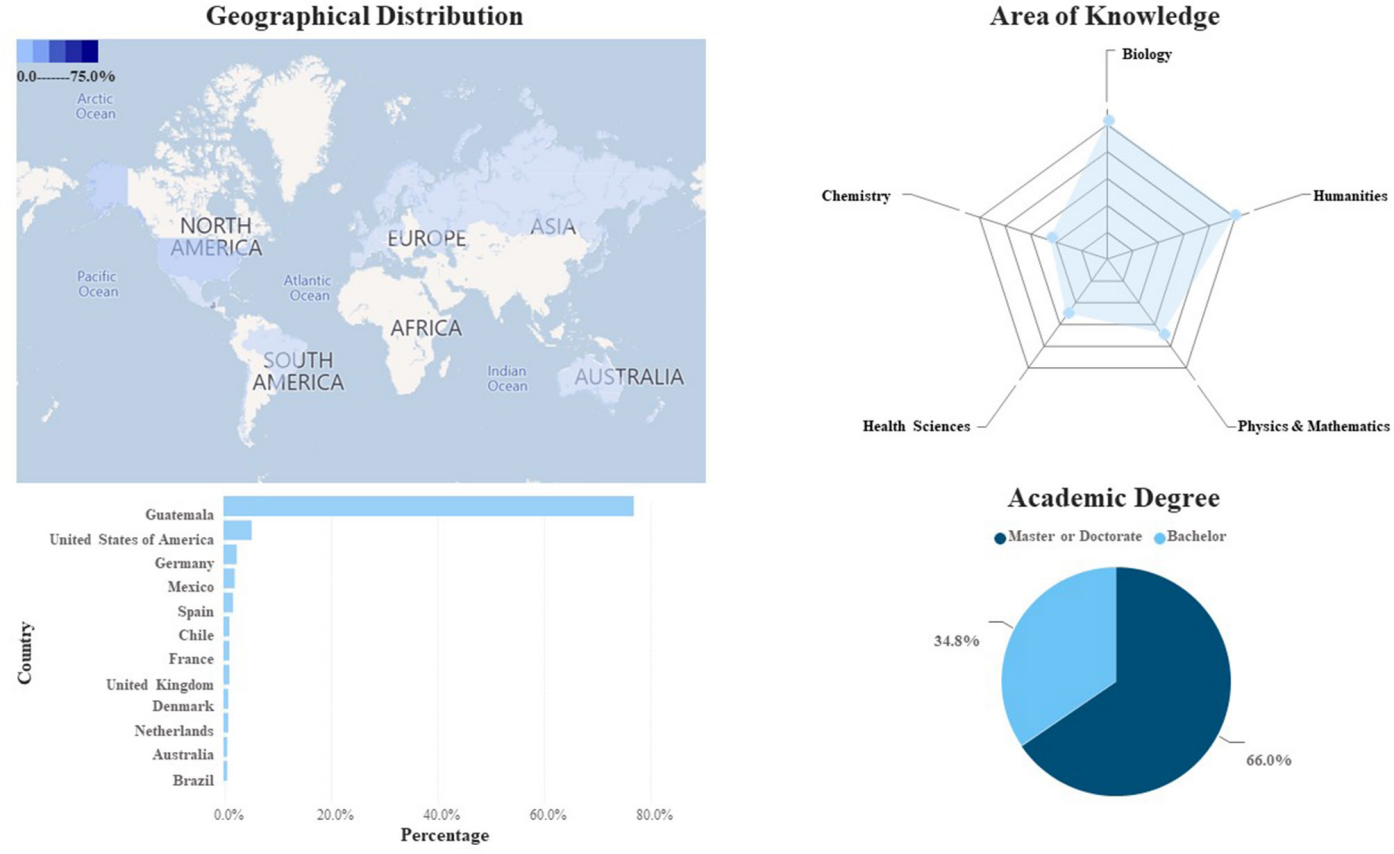
Geographical distribution, area of knowledge, gender, and academic degree distribution of members of the Guatemala National Chapter of the Organization of Women in Science for the Developing World OWSD (February 2022).

OWSD-Guatemala is a community of women scientists conforming to the national section of the global organization Women in Science for the Developing World (OWSD). This international organization was co-founded by UNESCO and the World Academy of Sciences TWAS in 1989 (OWSD, 2021). OWSD operates in four regions: 1. Africa, 2. The Arab Countries, 3. Asia-Pacific, and 4. Latin America and the Caribbean (LAC). OWSD-Guatemala, as a national section, belongs to the LAC region. To establish a national section (country-based), a minimum of 20 members must apply for international recognition supported by a local organization (Host Institution) with legal status and active operations. In the case of OWSD-Guatemala, the National Chapter is hosted by the Academy of Medical, Physical, and Natural Sciences (OWSD, 2020). As of February 2022, this network incorporated over 435 women scientists. The network's website allows the identification and location of members by name, area of expertise, year of membership, and OWSD awards or fellowships. From the available data, 77.2 % (*n* = 336) of its members indicated they have a residence in Guatemala. Of those reporting living abroad, 11.3% (*n* = 49) indicated living in European countries, while 7.4% (*n* = 32) in countries of North America. 2.1% (*n* = 9) reports living in South America, 0.7% (*n* = 3) in Asia and just 0.9% (*n* = 4) in Oceania (incl. Australia). With regards to their area of knowledge, the proportion of self-reported disciplines were: 27.0% (*n* = 118) in Agricultural, Biology, Earth Sciences (incl. Space Sciences and Astronomy) as well as for Interdisciplinary, Humanities, Social and Economic Sciences, respectively; 19.3% (*n* = 84) in Astrophysics, Physics, Math, Engineering, Computer Sciences, and Communication, 14.0% (*n* = 61) in Veterinary, Livestock and Health Sciences and 12.0% (*n* = 54) in Chemistry and Food Sciences. In terms of the level of education, 34.8% (*n* = 148) hold academic degrees at the undergraduate level, while 66.0% (*n* = 287) hold academic degrees at the graduate level (master's or doctoral) (Figure 3).

### Interaction Between the Scientific Diasporas and Stakeholders in Guatemala

Interactions between the GSD and different stakeholders seem to be erratic, episodic, and promoted with fragmented initiatives that ultimately obtain partial and inconsistent results. Moreover, as the engagement of the GSD with their country of origin is still unexplored to a significant extent, the roles and responsibilities among key stakeholders in their interaction with GSD is still a pending issue.

**Figure 4:**
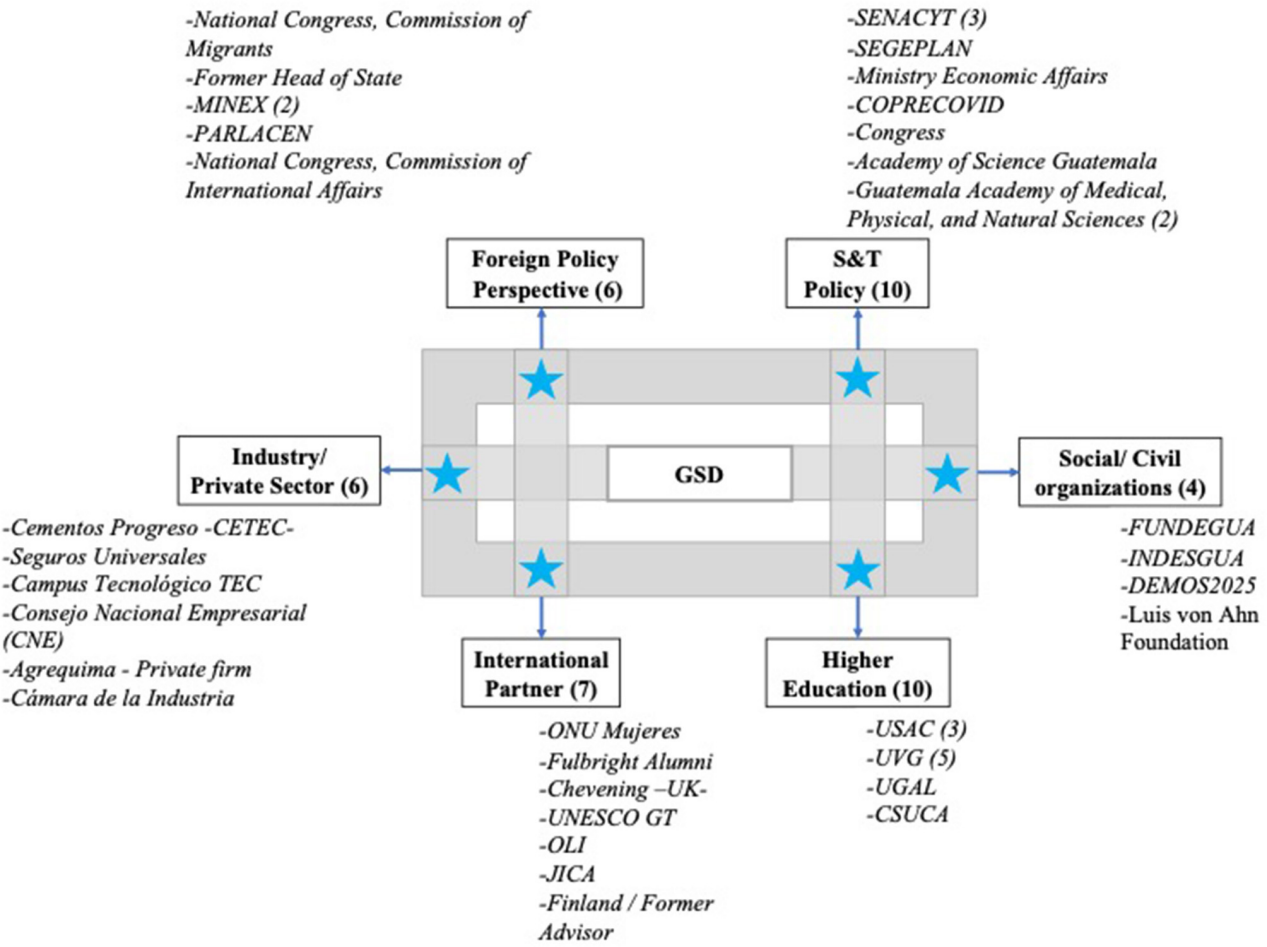
Participant organizations representing the distinct sectoral stakeholders relevant for mapping the GSD (September–February 2022). S&T, Science and Technology; GSD, Guatemalan Scientific Diaspora; MINEX, Ministry of Foreign Affairs; PARLACEN, Central American Parliament; SENACYT, National Secretary of Science and Technology; SEGEPLAN, National Secretariat of Planning; COPRECOVID, National Commission for COVID19; TEC, Technology Campus; OLI, Online Learning Initiative; JICA, Japanese International Cooperation Agency; USAC, University of San Carlos of Guatemala; UVG, University Del Valle; UGAL, University Galileo; CSUCA, Secretary General of the Central American University Council; FUNDEGUA, Guatemala Foundation, INDESGUA, Institute for the Development of Higher Education in Guatemala.

At the policy level, CONCYT is the governing structure in scientific and technological development in the country. It is responsible for the promotion and coordination of activities carried out by the National System of Science and Technology members. The Law for the Promotion of National Scientific and Technological Development (Congreso de la República de Guatemala, 1991) establishes that CONCYT is the country's highest decision-making and policy creation institution. This body coordinates entities relevant to Guatemala's science and technology areas, including those in the industry and academic sectors. In other words, CONCYT oversees national scientific and technological development. Nine members integrate this council; the Vice-President of Guatemala (who Chairs the CONCYT), the Minister of Economy, the President of the Science and Technology Commission in the Congress of Guatemala, the President of the Chamber of Industry, the President of the Chamber of Agribusiness, The President of the Chamber of Commerce, The Rector of San Carlos University (the public University), a representative on behalf of the rectors of all private universities and the President of the Academy of Medical, Physical and Natural Sciences of Guatemala. This council leads the country's national policies related to science and technology, but the functional part is delegated, by law, to SENACYT. As part of this study, the research team mobilized critical stakeholders from six perspectives: Science and technology policy, foreign policy, industry/private sector, civil society organizations, higher education, and international cooperation organizations (Figure 4).

## Discussion and Findings

**Figure 5:**
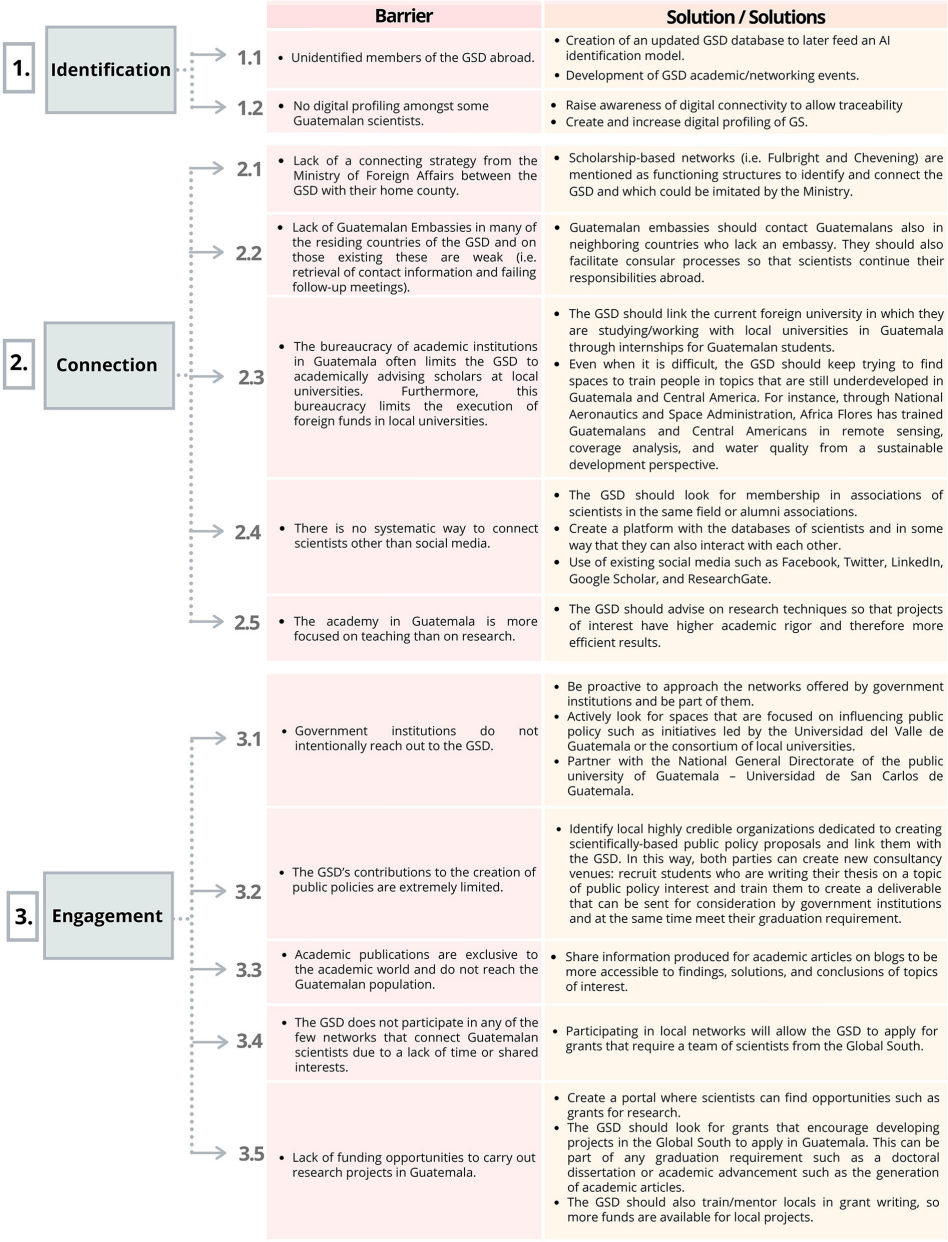
Guatemalan Scientific Diaspora (GSD) - Barriers and solutions identified by members of the GSD (September 2019-February 2020).

To facilitate the discussion and in harmony with the design of the research instruments, three stages are laid out in this section, considering the process of engaging the GSD with their country of origin. The first stage—Identification—involves the very existence of a GSD, the sense of belonging on behalf of the scientific community, while grasping the understanding of GSD from the key stakeholders. In this stage, it is critical to gain in-depth knowledge of the composition and characteristics of the GSD. The second stage -Connection—reflects the results obtained from the conversations between the GSD and their engagement in Guatemalan activities related to conferences, research projects, and others. The third stage -Engagement- is related to policy and sustained actions through which the GSD can effectively exert positive and constructive influence back on Guatemala. The participants in each stage identified barriers and obstacles and proposed recommendations and suggestions to overcome such challenges. The three stages are portrayed in the framework for the data analysis framework presented in Table 6 and Figure 5.

**Table 6:** Framework for data analysis—stages in engaging with the GSD.

**Stage**	**Guiding questions**	**Means/mechanisms**
Identification	What is a scientific diaspora? Who becomes a member of the Guatemalan Scientific Diasporas (GSD)? Where is the GSD located (countries of residence? In which fields of knowledge do the GSD members do research? Which characteristics (gender, level of education) do the GSD present?	Networking platforms Existing structured mechanisms (beyond social media groups) Systematic Group Participation
Connection	How do the members of the GSD interact among them? How do the members of the GSD interact with other stakeholders?	Social Networks (e.g., LinkedIn, ResearchGate), Alumni Associations (e.g., Association of Guatemala ex-Fulbright scholars, DAAD Alumni, KOICA Alumni) Individual initiatives (informal groups) Actions promoted by institutions organizations (e.g., INDESGUA—interactions among scholarship awardees, *Seguros Universales*—*Guatemaltecos Ilustres*)
Engagement	Which types of engagement have been experienced by the GSD? Which forms of engagement have proven effective/ineffective? What are the obstacles for the GSD engagement? Which solutions can be identified to overcome the obstacles to the GSD engagement with their country of origin?	Isolated Activities/Events Legislative actions Science and Technology Policy Foreign Policy Artificial Intelligence Machine Learning Policy and practice—institutions/organizations reached out to the GSD

### Identification of the GSD

#### Scientific Community

Most of the participating scientists clearly understood the term *diaspora*; however, they pointed out that it was not commonly used in reference to Guatemalan general emigration and rarely apply to the relocation of Guatemalan researchers abroad. In addition, while some participants declared understanding the term *scientific diaspora* as “structured and organized groups of people interested in science who work and study abroad”, others related the term to “unstructured, dispersed groups of scientists around the world”. Participants indicated they clearly understood the concept *scientific diaspora* after searching for its meaning in dictionaries and expressed having identified themselves with the concept. The most common answer for the GSD interviewees on the reasons for migrating was the lack of academic opportunities in their scientific area of interest and the professional development barriers in Guatemala. Nearly all participants related scientific diasporas with brain drain.

Evidence collected in this study suggests diaspora is not a common term in the academic Guatemalan language. It was exposed that most Guatemalan scientists felt identified with the characteristics of the SD, thus they felt as part of the scientific diaspora from the focus groups. Nevertheless, many of the participants mentioned it was until recently learned the meaning of this term. The host institutions also expressed that it was a new term. A recent increase of conferences from Guatemalan scientists promoting this topic has created an effect on scientific diaspora awareness. For example, SENACYT in Guatemala has recently incorporated issues related to the importance of the scientific diaspora after promoting conferences and events^3^.

Members of the GSD mentioned only three platforms to identify and map other Guatemalan scientists from the diaspora: the International Network of Science, Technology, and Innovation of Guatemala (RedCTI), the National Chapter for Guatemala of the Organization of Women in Science for the Developing World (OWSD-Guatemala), and the National Directory of Researchers -DNI- created by the Guatemala Nacional Secretariat of Science and Technology (SENACYT). The DNI is the most extensive database of researchers in the country; however, the GSD indicated they have heard about it in general terms, yet it does not include specific targets for Guatemalan scientists residing abroad. The DNI platform has filters, statistics, and promotion limitations to include more scientists living abroad. According to sustained communications with the technical personnel from SENACYT during the study, this platform will be improved through 2022. Changes include improving a more pleasant user experience, updating profiles, and promoting the site to increase the number of registered scientists.

Participants see an opportunity to build a robust platform using the technology and the data available in the existing platforms (RedCTI, OWSD-Guatemala, and DNI), similarly to experiences of other countries from the region and through the application of technological tools. For example, Costa Rica has a scientific diaspora mapping tool on the web with information about current location, plans of return to the government, reasons to leave the country, and collaborations opportunities^4^. Recent research (Loannidis et al., 2022) has shown that mapping and characterization of the scientist diaspora could be done using search engines and authorship directories in journals such as Scopus. For this, a specific question from the instrument used in this study explored the possibility and potential of technology such as artificial intelligence (AI) and even machine learning to identify Guatemalan items and, thus, aid in mapping its diaspora. AI can remain a tool to produce valuable information that impulses essential public policies and government plans to integrate the GSD with their national counterparts. Among the obtained ideas, the possibility of mining data from Guatemalan scientists' articles for repositories could support the generation of monthly newsletters to engage the GSD, as already explored for the Greek Scientific Diaspora (Loannidis et al., 2022).

Some participants considered that through AI and machine learning, the process of developing algorithms and statistical models could be adapted to identify and analyze patterns in the GSD. Mainly as more researchers are familiar with social networks, informing systems with information about their country of origin, institutional affiliation, and fields of research. Conversely, various participants consider that AI or machine learning might not be applicable to identifying, mapping, and characterizing the GSD as this is not large enough to require such complex technology. In any case, participants agree that the current state of the GSD shows inefficient articulation, management, and identification of the potential of the GSD.

#### Key Stakeholders

Most stakeholders understand the concept of scientific diaspora. They frequently refer to it as a loss for the emigrating country caused by poor conditions in the emigrating country regarding resources, technology, education, and political and economic stability. For example, civil society stakeholders reflected on the loss of connection between the diaspora and their home country. However, stakeholders in the industry expressed their understanding of the diaspora with a positive sentiment, highlighting how it represents new opportunities for migrating scientists and new opportunities for collaborations between the industry and the GSD. While still understanding the concept of diaspora as a loss in human capital, stakeholders representing international partners also expressed it as a possibility to strengthen Guatemalan systems, as one of them shared: “I thought of seeds that sprout for science or the beginning of strengthening a system.” Stakeholders representing the Science and Technology (S&T) policy sector pointed out that the definition of diaspora implies elements of organization and similar objectives, which does not characterize the GSD now. Notably, stakeholders representing Foreign Policy institutions were not entirely familiar with the concept of scientific diasporas.

In general, stakeholders indicated a lack of structured actions focused on mapping the GSD. Still, most could identify entities with the potential (and, perhaps, the responsibility). Stakeholders representing international partners expressed a keen interest in mapping the GSD and understanding the landscape regarding scientific development in Guatemala. An initial report about scientific development in Guatemala was published by UNESCO titled “Survey of research and innovation in the Republic of Guatemala” (Lemarchand, 2017), which is currently being updated in collaboration with SENACYT. Still, SENACYT, CONCYT, and Red CTI were named the existing networks with an untapped potential to map the Guatemalan diaspora. Representatives of higher education also see potential in the involvement of embassies. In general, OWSD was mentioned by most stakeholders as a network that is making a significant effort to collect data about women in the GSD and engage them in relevant topics of education and development in Guatemala. Notably, stakeholders in foreign policy institutions were unaware of any association or network to identify the GSD.

Stakeholders also identified examples of other functioning networks that have either successfully mapped a specific part of the GSD or could be used as a model for future efforts to map the GSD. Within these, alumni networks were often mentioned. Stakeholders in S&T indicated that alumni associations such as Taiwan and South Korea are perfect examples of strong networks. International partners also said the Chevening scholarship Alumni as a strong network with many members of the GSD. In relation to this, higher education representatives pointed out that communication between local scientists and scientists abroad is usually personal and based on personal interest or personal motivation. In line with this, they also identified the need for three essential elements to determine the GSD: “have people registered…then have them organized [through] an organization that allows them to interact, and finally institutionalization is needed, [meaning] someone who is in charge to follow up”. Representatives of higher education also seem to have a bigger picture of the importance of identifying human capital for the development of Guatemala. One of them mentioned, “we [Guatemalans] not only have the potential of Guatemalans abroad but also of scientists who are in foreign universities and whose field of work is Guatemala, which is a very great potential.”

Most stakeholders agreed that mapping the scientific diaspora is necessary and that a significant limitation in achieving this is the lack of a centralized effort to collect such information. Though most acknowledged the potential of AI to map the diaspora, few suggestions were made regarding its specific applications. International partners, for example, questioned what exactly would be considered AI while suggesting data mining to match publications, author names, and their countries to build a database of Guatemalan researchers and their work. Representatives of higher education identified the databases from *GuateFuturo* and SEGEPLAN as essential sources to identify the GSD while suggesting that the use of AI should be left for later. That initial effort should focus on constructing robust databases—an idea also shared by stakeholders in S&T.

### Connection With the GSD

The National Development Plan, *K'atun*, Our Guatemala 2032 (Conadur/Segeplan, 2014) includes Guatemala's section in the international development agenda. This plan delimitates the need for Guatemala to redesign its development model, promoting bilateral, regional, and multilateral relations to adapt to the demanding challenges of a changing world. Emphasis is placed on the interactions between Government, civil society, and international partners, which should intensify and diversify to establish clear roles and responsibilities for each actor to contribute to the development of Guatemala. This certainly includes a mature and consistent foreign policy. The plan, however, does not have any concrete reference to policy or actions to facilitate the engagement with the GSD. It only mentions efforts to reduce poverty and irregular (low-skilled and vulnerable) migration; the plan does not address the high-skilled migration. As for the state of science and technology in Guatemala, a general section is included in the document describing the precariousness of these sectors in terms of investment and capacity building (Conadur/Segeplan, 2014). Yet again, no explicit mention of the GSD is found. The General Government Policy 2020–2024 (SEGEPLAN, 2020) presented in the Planning and Programming Secretariat of the Presidency, SEGEPLAN, includes a chapter about migrants, remittances and human rights protection. The *K'atun* Plan does not provide additional information about GSD or actions to reduce the brain drain.

#### Scientific Diaspora

In general, the GSD expresses its willingness to contribute to the country's development; however, several members of the GSD still feel disconnected from Guatemala. Many factors influence this feeling. One that prevails is the lack of intentionality and action by governmental authorities. The lack of a governmental strategy, structured cooperation/interaction mechanisms, nor an intentional approach to the Guatemalan scientists is finely expressed by one scientist:

*And at the national level, it is difficult to engage because your very own Nation does not contact you to know where you are or what you are doing […]. We have the knowledge and the desire to contribute to the country, but we don't see where, if we don't look, we make the effort to see where, right, if the government itself doesn't contact us, it's not interested, it doesn't know we exist*. -Member of the GSD

The SENACYT was the most frequently mentioned institution to connect the GSD with Guatemala regarding governmental responsibility. In this respect, it is relevant to acknowledge that this institution was created as an executive body to implement the STI public policies emanated from the CONCYT. With the current governing structure of S&T in Guatemala, CONCYT is the body in charge of designing and issuing policy guidelines in these sectors, while SENACYT merely implements such policies. In this sense, SENACYT officers participating in this study acknowledge the institution's role as a significant stakeholder; however, they call attention to the limitations. Particularly regarding budget allocation, which is low and undermines SENACYT's capacities to achieve its objectives, the trends in the financial resources allocated to the institutions have suffered steady reductions. SENACYT reports 59 employees as permanent staff, while 23 provide services holding temporary employment (SENACYT, 2022), which in addition to the budget limits (< US$5 million a year), negatively affects its capacities (MINFIN, 2021).

Besides governmental support, the primary barriers connected with the GSD include (a) The lack of doctorate programs in the country, which prevents more robust platforms from linking with local researchers. Moreover, social and economic scientists consider it essential to highlight the financial contributions of science to connect with government stakeholders and the population. (b) Lack of time was a barrier linking side projects in Guatemala or participating in existing networks. One of the participants mentioned, “Carrying out research takes a long time in collaboration, 5–6 years”. (c) The lack of funds incentivizes locals to connect with the GSD and academic institutions, where teaching has more weight than research.

The members of the GSD suggested the use of existing technology platforms to connect with different projects and locals in Guatemala. Most mentioned platforms were WhatsApp®, email, video conferencing platforms such as Google Meets, Microsoft Teams, Zoom, Blue Jeans; social networking platforms such as private groups and public αMeta (Facebook) pages. They mentioned that using the existing ones could prevent social media exhaustion. Some others proposed the creation of new platforms that could allow for “posting” projects/interests, so the GSD can easily connect and identify opportunities for collaboration with other Guatemalan scientists, professionals, and other national authorities. Using AI algorithms, data can be mined, joined, and categorized to identify matching interests and demand of skills.

#### Key Stakeholders

Stakeholders are aware of numerous initiatives in the academic and scientific communities that promote and enable collaboration with scientists of the GSD. They acknowledged that it is mostly by individual researchers' motivation and through their personal and professional network that a large part of the existing collaborations is created. The strengthening of existing institutional networks was strongly recommended to promote working relationships between scientific communities and the GSD. Likewise, some specific scholarship programs, such as the German and UK Academic Exchange Service (DAAD and Chevening, respectively), were named influential in providing scholarships for higher education and maintaining strong ties and ongoing communications with its recipients.

Technology was mentioned as a valuable tool to facilitate communication and exchange between both parties. For example, using Zoom, Google Meets, or YouTube to host conferences and talks where researchers can present their work and highlight opportunities for collaboration. ResearchGate, Academia, LinkedIn, Twitter, and Facebook groups were also recommended as a tool that allows researchers to meet one another and share their profiles and expertise. However, some stakeholders expressed concern over the excess of existing social and professional platforms and proposed that a new platform would require powerful incentives for scientists to join.

Additionally, many thought it was critical to involve institutions such as CONCYT, SENACYT, and professional associations to make it easier to establish such connections and offer opportunities such as scholarships, research funding, and workshops for specialized training. Representatives of higher education identified some opportunities for improvement in CONCYT. They concluded that CONCYT needs to “keep a record, be active, have more budget, be more linked to GSD and include social sciences in its agenda.”

Interestingly, there are already some organized efforts between stakeholders and the GSD. Stakeholders in Civil Society indicated they already hold strong ties with the GSD, particularly involving scientific collaboration and support to students aspiring to receive specific scholarships or study in a foreign country. International partners also mentioned collaborating with the GSD, specifically on topics regarding education. They also expressed the desire to further support women in the GSD, noting a disparity in their available opportunities.

Representatives from different groups in society also identified some barriers to connecting the GSD with local scientific communities. Stakeholders in S&T policy mentioned that one of the significant challenges is the lack of interest of the GSD in working in the country. A participant noted, “Many of those scientists who live, and work abroad consider their condition of skilled emigrants as an achievement, [they] do not look back, they see escaping from their country as an opportunity in their career development. They are no longer interested in what happens back in their country; they may be working on a fascinating topic. Still, suppose they are asked to take part in collaborations. In that case, they disregard the invitations”. This sentiment was echoed by stakeholders in Civil Society, who mentioned that, once students' goal of moving abroad was achieved, they showed little interest in keeping in contact and little commitment to being involved in supporting projects in Guatemala.

Moreover, one of the higher education representatives said that “the main barrier that I see is mistrust and the few spaces for advocacy with the public sector”; there are many difficulties connecting with decision-makers and promoting initiatives. It is generally complicated for scientists to be heard in political spheres. One of them mentioned that connecting the GSD to the political sphere is very “ambitious,” yet believes that something more feasible is strengthening the GSD's connection with academic institutions to achieve better doctoral programs and research projects.

### Engagement of the GSD (Actions/Policies)

#### Scientific Community

The stage of Engagement refers to a systematic, constant, and sustained participation of the GSD in different schemes, mechanisms, actions, and policies through which they exert influence in their country of origin.

*Development must be driven by both the public [sector] and the private [sector]. A national policy to engage the GSD is necessary*. Member of the GSD

*The biggest problem is that at the national level, there is no policy to include scientists in the development process of our home country*. Member of the GSD

Regarding the actions developed by the Ministry of Foreign Affairs, the GSD has not identified any program that links diaspora scientists with the country, and even when the scientists have suggested collaboration options, they have not received any formal response. The GSD stated that some embassies are helpful for students who study abroad. The embassy of the United States of America in Guatemala and the embassy of the United Kingdom in Guatemala were identified as some of the most dynamic connections with their alumni. These connections might be more systematic as they offer prestigious and globally recognized scholarships such as the Fulbright and Chevening programs. The GSD also pointed out that another limitation is the lack of diplomatic missions in certain countries such as Hungary and Switzerland. Whereas, having a Guatemalan embassy near them, such as the case of the Guatemalan embassy in Korea, might provide more support and resources. Many other members of the scientific diaspora point out that the inefficiency in general bureaucratic processes translates into the inefficiency of the embassies in connecting with the GSD.

Regarding the interactions with the government, the GSD generally showed dissatisfaction with the work of government institutions for linking them with projects in Guatemala. They indicated that the government should be more intentional and proactive in contacting Guatemalan scientists abroad since the GSD “.... has the knowledge and desire to contribute to the country, but there are no clear ways to do it”. The GSD indicated that the meetings held by these institutions are fruitless because no concrete conclusions have been reached. It was also mentioned that the Red CTI is not agile. The research of institutions such as the Institute for Nutrition of Central America and Panama (INCAP) is not transferred to decision-makers. The GSD proposes that scientists get involved in the science and technology committees of the Guatemalan Congress. The GSD identified the importance of linking with the Ministry of Economy with structured conferences developed in Guatemala to involve more scientists. One of the actions suggested to get more involvement from the public sector is to measure indicators of the current situation in the country to assess the importance of making science-based decisions. Suggestions are used to strengthen the link between the Ministry of Economy and CONCYT, showing the economic relevance of science in the country to gain more confidence in the industrial sector with academia. The diaspora thinks that SENACYT has developed good outreach work, especially promoting STEM careers for girls. Among the limitations observed in the existing programs is the bureaucracy of the administrative system, i.e., for obtaining and managing funds, within the Red CTI meetings and decision-making without interaction with members, and lack of financial support for both research grants and follow-up of research activities.

They also mentioned the importance of *Converciencia*. This event in the past has provided opportunities for the GSD to connect with peers (period 2005–2020). The initiatives of the OWSD Guatemala chapter (period 2020–2021) have also been mentioned as crucial examples of how to link the diaspora and generate results and actions. Universities and industries have approached the diaspora collaborations by having them in conferences that have allowed them to connect and outreach to the Guatemalan audience.

Regarding the barriers to linking actions, scientists consider the lack of trust to give preference to their interests by the different sectors, the weak link between the academic and public sectors, the lack of public investment in research, development, and innovation, and the low value of science reflected in the workload and working conditions of most scientists residing in the country. A few scientists consider that top-down changes are complex and must be motivated from the bottom up, starting with transforming students' lives by researching and producing results that allow them to demonstrate the importance of science to the daily lives of the general population. One of the solutions AI could generate to support the actions for the GSD connections is to create a Guatemalan platform for the community of scientists. They proposed a tool that visualizes scientists' profiles and connects them directly with private institutions, government, or civil sector organizations interested in their academic contribution and professional skills.

Figure 5 summarizes the barriers and solutions identified by the Scientific Community, in the three stages.

#### Key Stakeholders

Stakeholders described current ties with the GSD as insufficient and weak. For example, stakeholders in the industry mentioned having some relations with the GSD but acknowledged these ties are weak and limited. Higher Education critiqued the approach of *Converciencia*, which members of the GSD identified as a valuable opportunity for connection. According to stakeholders in Higher Education, *Converciencia* was identified as a relevant mechanism for developing collaborations with the GSD. However, it shows at least two limitations (1) bringing scientists (including members of the GSD) who want to implement projects that do not correspond to the national context and (2) bringing scientists (including members of the GSD) who have prejudices about the knowledge of local scientists.

When identifying the reason for the current lack of collaborations, stakeholders underlined a lack of structure as one crucial obstacle to engaging the GSD in Guatemala's sustainable development. Stakeholders expressed that there is a lack of institutional systems and policies that enable the engagement of the GSD in the country's development. Only a few stakeholders had any knowledge about actions by the Ministry of Foreign Affairs and diplomatic corps in supporting such collaboration, which was described as insufficient and sporadic, even though both were considered relevant agents. Namely, the Department of State and the UK embassy were explicitly identified as entities that offer or have offered support to establish collaboration networks with the GSD. Likewise, stakeholders considered that there had been little action involving the GSD in topics of development and policymaking and a lack of interest and investment in science overall. SENACYT was mentioned as one of the most active government institutions because it finances the salaries of former members of the GSD who return to the country, involves GSD members in their activities, and has its Red CTI network. CONCYT was also mentioned as a relevant actor, although the result of its actions was deemed unsatisfactory. Finally, stakeholders representing Foreign Policy institutions considered that the Central American Parliament (PARLACEN) has the potential to generate regional impact in establishing such connections. However, its only action in this area is the creation of a regional fund for science and technology, which is currently being developed. The involvement of scientific experts in the development of industry was acknowledged as necessary. Still, none of the participating stakeholders had any knowledge of specific mechanisms that enabled the involvement of the GSD. It was generally agreed that universities and individual researchers are the main drivers of scientific collaboration with the GSD, using their personal and professional contacts to establish networks and develop projects.

A second significant obstacle to engaging the GSD in Guatemala's sustainable development was a lack of sufficient resources and incentives. For example, representatives of Higher Education identified two main barriers to connecting the GSD with local scientists: lack of time and lack of funding. It was mainly acknowledged that the scientific community deals with various limitations (e.g., time, resources, bureaucracy, and career demands) that hinder the support they can potentially provide. Furthermore, stakeholders in Civil Society observe a lack of institutional support for research and scientific development in Guatemala, which has resulted in a lack of opportunities and incentives for the GSD to be involved in actions for development in Guatemala, either by returning to the country or collaborating from abroad. This effect was perceived by industry stakeholders as a lack of interest from the GSD to be involved in projects in Guatemala and a tendency to “forget” about their home country. As one member said, “We have [a relationship with the GSD], but it isn't stronger because some of them have forgotten about Guatemala.” However, they acknowledged giving more support and incentivizing scientific development.

Stakeholders broadly commented on how the development of solutions should involve conjunct work between the public sector, private sector, and academia. However, some expressed concern over the lack of interest of government agencies in science and indicated that academia has the most substantial potential to lead the way in developing opportunities to involve the GSD in development projects. Still, all stakeholders could identify opportunities in which collaboration with the GSD would be fruitful. Particularly, stakeholders in S&T highlighted the importance of linking the GSD with the academic sector and the private and public sectors to develop such opportunities. In this way, the transfer of knowledge and technology could specifically target problems in Guatemalan society. Further, industry stakeholders reiterated their willingness to explore possibilities for collaboration with members of the GSD.

Regarding possible actions to overcome current obstacles, the need for more communication was highlighted. Industry stakeholders suggested having more open communication of industry needs to help identify opportunities for collaboration. Likewise, they underscored the importance of scientists showcasing their work and communicating their findings in a understandably and attractively way to the industry sector. One S&T stakeholder pointed out that multiple cultural barriers must be surpassed within the Guatemalan context, which requires contextualization and translation of scientific information.

**Figure 6:**
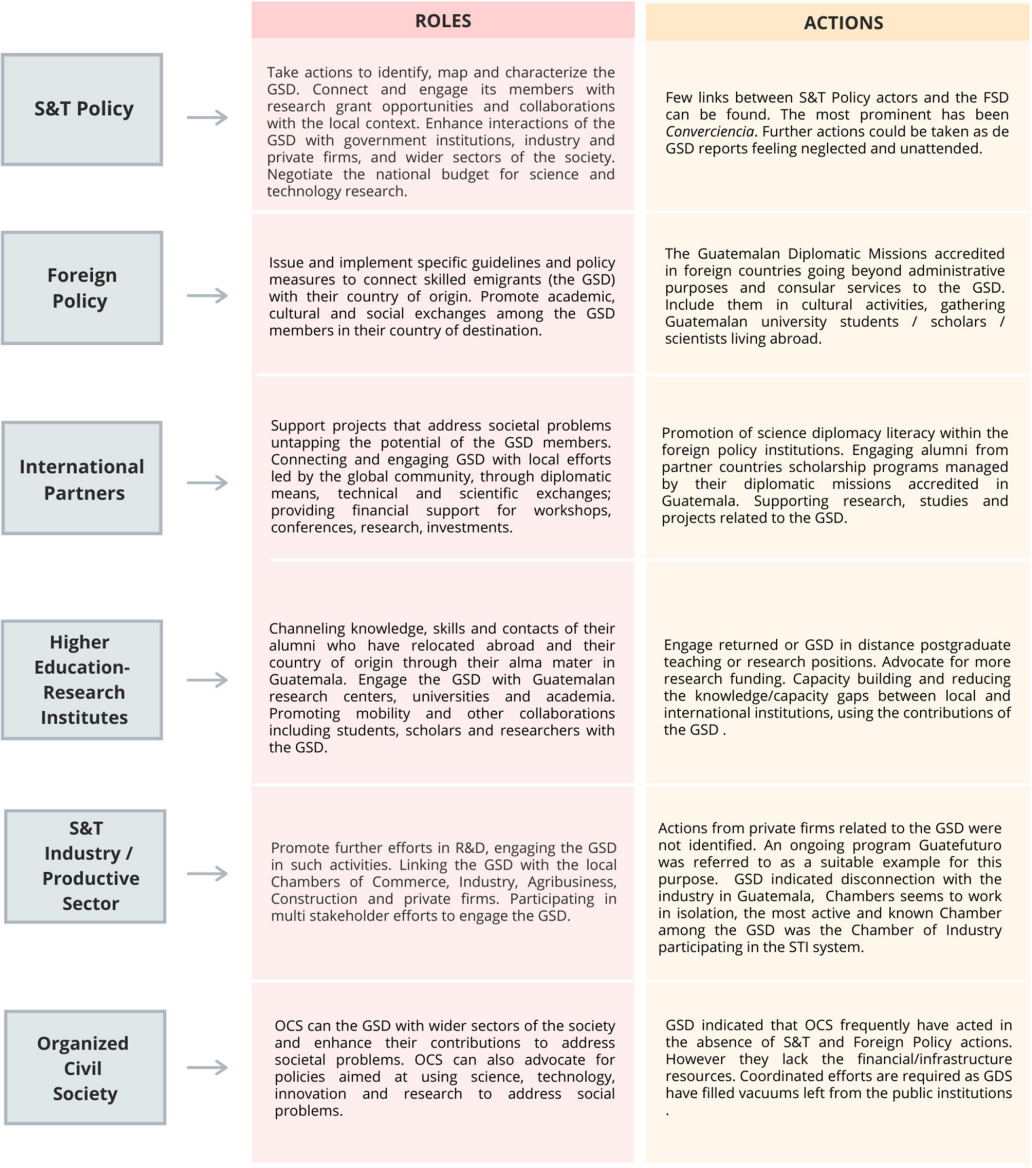
Stakeholders—roles and actions identified by participants in engaging the GSD (September 2019-February 2020).

Stakeholders also considered that governmental institutions need to be active in involving the GSD and that science policy needs to be developed—especially by linking it to economic policy and development to add incentives. Stakeholders in the S&T policy indicated that better inter-institutional organizations could create the connection of the GSD with projects in Guatemala. For example, they mention the communication between SEGEPLAN and SENACYT, where SEGEPLAN (in charge of administering international cooperation in the form of scholarships/fellowships) should systematically communicate the names and detailed information scholarship/fellowships awardees, especially at the graduate level (masters, doctoral programs). Then SENACYT can approach and connect them. Participants also mentioned the relevance of science diplomacy (as the interface between science and foreign policy) by suggesting that “the “SENACYT's policy and actions combined with the Ministry of Foreign Affairs [should engage] the GSD [by] having a scientific *attaché* in [Guatemalan] embassies and consulates, [and also] to train MINEX [Ministry of Foreign Affairs] staff and other public officials in science diplomacy guidelines.” Some of them also recommended having at least a minimum plan of things that scientists could contribute; in this way, they would guide them on how to apply their knowledge in the country. Figure 6 summarizes the roles and actions identified by stakeholders in engaging the GSD.

## Conclusions

To our knowledge, this study is the first to provide supportive evidence of the growing and existing community of scientists outside of Guatemala, namely the Guatemalan Scientific Diaspora (GSD). Members of such GSD shared their past experiences, attempts, and results of their efforts for engagement with Guatemala and their current interest in contributing to the country's development through specific actions that need to be coordinated between the distinct sectors: government, academia, and industry. The importance of mapping, characterizing, and understanding the GSD to have a strong capacity-building mechanism and networking between GSD and local actors also became apparent. Until today, the existence of a GSD has not been systematically identified, registered, or studied and remains an untapped resource for development in the country. Moreover, the GSD has made positive initiatives and efforts, although they lack a legal or operational framework. As an initial step, systematized baseline information is required to develop further the actions toward structuring the GSD, develop future policies aimed to engage them, and highlight their impact at multiple levels of the country of origin.

This study highlighted the lack of knowledge of the existence of a GSD, their interaction with their national counterparts and local stakeholders, and the need to recognize it and develop a structure or plan for them to interact efficiently with Guatemala. Due to the lack of articulation, independent and few successful experiences were shared by the participants of this study, though they recognized the potential to develop more substantial collaborations.

Numerous members of the scientific diaspora have also acknowledged their responsibility to seek opportunities to remain relevant and positively influence Guatemala. Although the ideal scenario would involve an organized and systematized collective action, individual initiatives are also valuable. A sense of responsibility was also repeatedly mentioned, as many GSDs have benefited from scholarships, fellowships, grants, and funding based on their nationality or country of origin.

In turn, our participants sensed a more positive view of the GSD and the perception of the host country. Based on our results, the continuation of collaboration with and employment of Guatemalan researchers was perceived as positive and possible. As stated by the subjects interviewed, skilled Guatemalan scientists contribute to furthering research on relevant topics, advance technological developments, allow deeper labor market specialization, and have a robust understanding of evidence-based knowledge and its application to real-world problems. Moreover, it was identified that the GSD allows the representation of an additional aspect of Guatemala: that of highly skilled, hard-working professionals who are passionate about science, technology, and development and make valuable contributions in their field.

Scientific diasporas are fundamental for science and research capacity development. They are also recognized as a solid force to encourage novel and fruitful collaborations abroad; therefore, governments, civil societies, other organizations, corporations, and academic groups are needed. In the case of Guatemala, no public policies or legislative actions, nor existing collaborative structures focused on creating possibilities of engagement with GSD were identified. In terms of Foreign Policy, attention is mainly centered on irregular and vulnerable forced emigration, particularly toward the United States of America. As for public policies in science and research, the single initiative identified as a consistent activity to connect GSD with their country was *Converciencia*, which is not a program nor a policy, but an event or recurrent activity (over 15 years of implementation with changes over time).

Evidence suggests that the GSD cooperates with governmental institutions such as the National Secretary of Science in established programs such as *Converciencia* or the International Guatemalan Scientists Network. Nevertheless, they complain about the lack of policies, bureaucracy, or non-existence of engagement programs. This is the best scenario. The other governmental institutions hadn't created any program or platform in most cases. The GSD has not found support in the Ministry of Foreign Affairs or Embassies, among other government institutions.

Guatemalan universities and research centers have a fundamental role in developing new strategies to increase inclusiveness and actively engage the GSD by responding to contemporary science's global demands and needs as part of their programs. They need to expand local scientists' participation by informing them about existing programs and promoting and sharing authority in engaging them in all the research decision-making processes. The inclusion of GSD is an opportunity for creating models of governance of science centers based on the participation of local and abroad scientists' experiences as integral components alongside the ones who traditionally place the role of these centers as a provider of trained persons and basic knowledge.

The GSD suggested strategies using artificial intelligence and machine learning to data-mine all the Guatemalan scientists' online professional information and publications. Members of the GSD also suggested creating a platform to enable better communication between the diaspora and the different key actors within the Guatemalan science, research, and innovation system. They indicated it is relevant to include actors and decision-makers from the government, higher education, research institutions, and the industry, relevant stakeholders representing international partners, non-governmental organizations, and civil organized social groups.

International partners play a relevant role in the engagement of the GSD, mainly through alumni associations, such as the Fulbright and Chevening alumni networks, having an active connection amongst alumni. International partners also provide research grants, scholarships for postgraduate studies, and short courses in their countries of origin. Other international (bilateral-multilateral initiatives) are also relevant to the GSD. They are sources of grants, awards, and spaces for researchers and scientists to connect and engage with their peers not only from their countries of origin but also from other regions with similar needs and challenges i.e., the International Network for Advancing Science and Policy -INASP-, the Organization of Women in Science for the Developing World-OWSD-, the World Academy of Science TWAS, the InterAcademy Partnership IAP, to cite some.

## Data Availability Statement

Publicly available datasets were analyzed in this study. This data can be found here: https://owsd.net/network/guatemala, https://redcti.senacyt.gob.gt/portal/index.php/investigadores/directoriocti.

## Ethics Statement

The studies involving human participants were reviewed and approved by the Ethics Committee from the University of Technology of El Salvador (UTEC). The patients/participants provided their written informed consent to participate in this study. All participants were asked to sign an electronic informed consent form prior to their participation.

## Author Contributions

KB: conceptualization (lead), data curation (lead), project administration (lead), methodology (lead), resources (lead), validation (lead), and writing—original draft (equal). CR-O and SA: conceptualization (equal), data curation (equal), supervision (lead), project administration (equal), writing original draft (equal), and visualization (equal). NYOO, MA, AD, and GO-C: investigation (supporting), visualization (supporting), writing original draft (equal), and writing—review and editing (equal). SM: formal analysis (equal), investigation (supporting), data curation (equal), and resources (equal). GM-B: critically analyzed the manuscript and suggested edits. NYOO: investigation (equal), data curation (equal), writing original draft (equal), and visualization (supporting). All authors contributed to the article and approved the submitted version.

## Funding

This research received funding from the International Network for Advancing Science and Policy (INASP) through the grant INASP0652021.

## Conflict of Interest

SA was employed by New Sun Road. The remaining authors declare that the research was conducted in the absence of any commercial or financial relationships that could be construed as a potential conflict of interest.

## Publisher's Note

All claims expressed in this article are solely those of the authors and do not necessarily represent those of their affiliated organizations, or those of the publisher, the editors and the reviewers. Any product that may be evaluated in this article, or claim that may be made by its manufacturer, is not guaranteed or endorsed by the publisher.

## Footnotes

^1^https://redcti.senacyt.gob.gt/portal/.

^2^https://www.senacyt.gob.gt/.

^3^See https://blog.inasp.info/guatemala-scientific-diasporas/ and https://www.youtube.com/watch?v=0qSvnehuU4M.

^4^https://hipatia.cr.
