# Supplementary material for: Engaging the Guatemala Scientific Diaspora: The Power of Networking and Shared Learning
Source: Front Res Metr Anal. 2022 Jun 8;7:897670. doi: 10.3389/frma.2022.897670 (PMC9215311; doi:10.3389/frma.2022.897670)
Supplement: Supplementary file 1 [file Data_Sheet_1.PDF]

Study: Engaging the Guatemala Science Diasporas for Development: Network building and shared learning

"Engaging the Guatemala Science Diasporas for Development: Network building and shared learning"

Focus Group A: Scientific community

Part I: Existence/Identification/Characterization/Mapping of the Guatemalan Science Diaspora

Question 1: Do you personally know of or have you heard of the existence of networks, groups, or identification mechanisms of the Guatemalan Science Diaspora?

- If positive answer, could you please share the mechanisms, actions or policies you know of?

- If negative answer, why do you think there is this lack?

Question 2: Do you think Artificial Intelligence (AI) could help with the mapping, identification or characterization of the Guatemalan Science Diaspora?

- If positive answer, can you please tell us how do you think AI can help?

- If answer is short/no answer, please provide some examples of how it has been used in other countries.

Part II: Engaging of the Guatemalan Science Diaspora with their country of Origin

Question 3: Epistemic community. (Groups of experts) During your career, have you collaborated with scientists or experts from Guatemala in the diaspora in any scientific project?

Question 4: Scientific Advice. Have you contributed (or do you know of any initiative that has contributed) with evidence-based scientific fundaments to the design, formulation, implementation or reform of public policies in Guatemala?

Question 5: Do you know of scientific projects or initiatives of other Guatemalans in the Diaspora that are currently being implemented and are related to science or the generation of knowledge in (or for) Guatemala?

If the answer is affirmative, do you know of any institution and/or network of members of the Guatemalan Science Diaspora who work together or collaborate on issues related to public policies or sustainable development projects in Guatemala?

Question 6: Based on your experience, do you know of actions or mechanisms that are part of Guatemalan foreign policy (through the Ministry of Foreign Affairs or Guatemalan Diplomatic Missions abroad) that seek to link you as a scientist with Guatemala?

Question 7: Have you used any technological platform that links your research to scientific communities within Guatemala?

- If the answer is positive, can you please share your experience with us?

Question 8: Do you consider it is importat to use technological platforms to connect the Guatemalan scientific diasporas and the different scientific and technological communities of the country?

If yes, what technological platforms do you recommend to link the Guatemalan scientific diasporas and the different scientific and technological communities of the country?

Part III: Policies/Practices/Channels for linking the Guatemalan Science Diaspora with the Development of Guatemala

Question 9: Have you been contacted or invited to participate in any group, channel or mechanism to connect the Guatemalan Science Diaspora?

- If yes, what benefits do you consider this type of network brings to the scientific community of the country?

Question 10: What technological tools could be useful to create mechanisms, policies and actions to involve the Guatemalan Science Diaspora with the development of Guatemala?

Question 11: How can knowledge generators (researchers) be connected to users of this knowledge (decision makers, policy makers, broad sectors of the Guatemalan population)? In other words, how can technology best contribute to the production and dissemination of knowledge relevant to the needs of society?

Question 12: Do you know any institution and/or networks of the Guatemalan Science Diaspora who work together or collaborate on issues related to public policies or sustainable development projects in Guatemala?

- If yes, what have these mechanisms and/or practices consisted of?

Part IV: Challenges/Barriers/Obstacles to creating Links

Question 13: What do you consider to be the greatest challenges or barriers to creating links with the Guatemalan scientists abroad?

Question 14: What initiatives or actions do you consider could increase the link with Guatemalan Science Diaspora?

Question 15: Do you know of another study/ies or have you heard of similar research or with similar themes/objectives to this one?

OWSD GUATEMALA
Organization for Women in Science for the Developing World

Question 16: Would you like to mention/recommend another person or networks that you think could contribute to this study?

Question 17: Do you have any additional comments/suggestions regarding this study?

Closing: Thank you very much for your participation. Please, do not hesitate to send any additional information at your convenience to the email address owsd.guatemala.chapter@gmail.com

Glossary of key terms: Understanding key terms in the context of this research

Machine learning: discipline in the field of Artificial Intelligence that, through algorithms, equips computers with the ability to identify patterns in big data and make predictions (predictive analysis).

Science Diaspora: Scientists, researchers, engineers, professionals with advanced studies, training and qualifications who reside outside their country of origin or participate in mobility mechanisms for extended periods, particularly in areas of science, technology and education.

Artificial Intelligence: Artificial Intelligence or AI, is a technology that, through a series of algorithms, gives a machine, processor or software the ability to interpret, decide and solve problems autonomously using the input it receives.

Epistemic community: Network or groups of professionals with recognized experience and competence in a particular field. Experts who, due to their specialized knowledge, have legitimacy in the policy area within a given field.

Scientific Advice: It is the process, structures and institutions through which governments, politicians and decision makers consider scientific information and evidence in making decisions and policies. Scientists provide scientific advice.

National Innovation System: Open system made up of organizations, institutions, individuals or communities of individuals in a country that influence the design, development, implementation of policies, strategies, programs, methodologies and mechanisms for the management, promotion, financing, protection and dissemination of scientific research and innovation

Technological Platforms: Mechanisms that facilitate the exchange of information, promote participation, and foster cooperation in applied research projects and technology development.
