# Supplementary material for: Engaging the Guatemala Scientific Diaspora: The Power of Networking and Shared Learning
Source: Front Res Metr Anal. 2022 Jun 8;7:897670. doi: 10.3389/frma.2022.897670 (PMC9215311; doi:10.3389/frma.2022.897670)
Supplement: Supplementary file 2 [file Data_Sheet_2.PDF]

Study: Engaging the Guatemala Science Diasporas for Development: Network building and shared learning

"Engaging the Guatemala Science Diasporas for Development: Network building and shared learning"

Semi-structured Interview A: Scientific Community

Date: __________ Name of interviewee:_____________________________________

Institution or affiliation:____

Name of interviewer: _______________ Interview mechanism _______________

Duration:___________ / For key terms in this interview please see the glossary at the end

Part 1: Career path of interviewee and their relationship to the topic of study

Question 1: What ideas, concepts or definitions come to mind with the term "Science Diaspora"?

Question 2: Please describe how your career path (studies, work experience) relate to the topic of the Guatemalan Science Diaspora?

Question 3: Would you like to share additional information about your career path and how you are linked to the Guatemalan Science Diaspora?

Part 2: Existence/Identification/Characterization/Mapping of the Guatemalan Science Diaspora

Question 4: Do you personally know of or have you heard of the existence of networks, groups, or identification mechanisms of the Guatemalan Science Diaspora?

- If yes, could you please share the mechanisms, actions or policies you know of?
- If no, why do you think there is this lack?

Question 5: Do you think Artificial Intelligence (AI) can help with the mapping, identification or characterization of the Guatemalan Science Diaspora?

- If yes, can you please tell us how do you think AI can help?

Part 3: Engaging of the Guatemalan Science Diaspora with their country of Origin

Question 6: Epistemic community. (Groups of experts) During your career, have you collaborated with scientists or experts from Guatemala in the diaspora in any scientific project?

Question 7: (Scientific Advice). Have you contributed (or do you know of any initiative that has contributed) with evidence-based scientific fundament to the design, formulation, implementation or reform of public policies in Guatemala?

Question 8: Do you know of scientific projects or initiatives of other Guatemalans in the Diaspora that are currently being implemented and are related to science or the generation of knowledge in (or for) Guatemala?

Question 9: Based on your experience, do you know of actions or mechanisms that are part of the Guatemalan foreign policy (through the Ministry of Foreign Affairs or Guatemalan Diplomatic Missions abroad) that seek to link you as a scientist with Guatemala through the Diplomatic Missions in your country of residency?

Question 10: In your experience, Have you had any engagement/participation with initiatives to strengthen the Guatemalan National Innovation System (SNI) (e.g. through universities, SENACYT, civil society organizations, foundations, etc)?

Question 11: How can technological platforms help to link the Guatemalan science diasporas and the different scientific and technological communities of the country?

Question 12: Which technological platforms do you recommend to link the Guatemalan science diasporas and the different scientific and technological communities of the country?

Question 13: Have you used any technological platform that links your research to scientific communities?

- If yes, can you please share your experience with us?

Part 4: Policies/Practices/Channels for linking the Guatemalan Science Diaspora with the Development of Guatemala

Question 14: How can knowledge generators (researchers) be engaged to users of this knowledge (decision makers, policy makers, broad sectors of the Guatemalan population)? In other words, how can technology best contribute to the production and dissemination of knowledge relevant to the needs of society?

Question 15: Do you know of any institution and/or network of the Guatemalan Science Diaspora who work together or collaborate on issues related to public policies or sustainable development projects in Guatemala?

- If yes, what have these mechanisms and/or practices consisted of?

Question 16: Have you been contacted or invited to participate in any group, channel or mechanism to engage with the Guatemalan Science Diaspora?

- If yes, what benefits do you consider this type of network brings to the scientific community of the country?

Question 17: What technological tools could be fundamental to create mechanisms, policies and actions to involve the Guatemalan Science Diaspora with the development of Guatemala?

Part 5: Challenges/Barriers/Obstacles to creating Links

Question 18: What do you consider to be the greatest challenges or barriers to creating links with the Guatemalan Science Diaspora?

Question 19: What initiatives or actions do you consider could reduce the barriers to creating connections with Guatemalan Science Diaspora?

Question 20: Do you know of another study/ies or have you heard of similar research or with similar themes/objectives this one?

Question 21: Would you like to mention/recommend another person or networks that you think could contribute to this study?

Question 22: Do you have any additional comments/suggestions regarding this study?

Closing: Thank you very much for your participation in this interview. We have taken ___ minutes to complete the interview, do not hesitate to send any additional information at your convenience to the email address owsd.guatemala.chapter@gmail.com

Glossary of key terms: Understanding key terms in the context of this research

Machine learning: discipline in the field of Artificial Intelligence that, through algorithms, equips computers with the ability to identify patterns in big data and make predictions (predictive analysis)

Science Diaspora: Scientists, researchers, engineers, professionals with advanced studies, training and qualifications who reside outside their country of origin or participate in mobility mechanisms for extended periods, particularly in areas of science, technology and education.

Artificial Intelligence: Artificial Intelligence or AI, is a technology that, through a series of algorithms, gives a machine, processor or software the ability to interpret, decide and solve problems autonomously using the input it receives.

Epistemic community: Network or groups of professionals with recognized experience and competence in a particular field. Experts who, due to their specialized knowledge, have legitimacy in the policy area within a given field.

Scientific Advice: It is the process, structures and institutions through which governments, politicians and decision makers consider scientific information and evidence in making decisions and policies. Scientists provide scientific advice.

National Innovation System: Open system made up of organizations, institutions, individuals or communities of individuals in a country that influence the design, development, implementation of policies, strategies, programs, methodologies and mechanisms for the management, promotion, financing, protection and dissemination of scientific research and innovation

T**echnological Platforms***:* Mechanisms that facilitate the exchange of information, promote participation, and foster cooperation in applied research projects and technology development
