# Supplementary material for: Engaging the Guatemala Scientific Diaspora: The Power of Networking and Shared Learning
Source: Front Res Metr Anal. 2022 Jun 8;7:897670. doi: 10.3389/frma.2022.897670 (PMC9215311; doi:10.3389/frma.2022.897670)
Supplement: Supplementary file 3 [file Data_Sheet_3.PDF]

Study: Engaging the Guatemala Science Diasporas for Development: Network building and shared learning

"Engaging the Guatemala Science Diasporas for Development: Network building and shared learning"

Focus group B Stakeholders (Perspectives)

Summary of Participant Profiles

Perspective	Profile of Stakeholders (Actors)
A - Science and Technology Policies	Institution/Organization relevant to Guatemala's science and technology policies e.g. SENACYT, SEGEPLAN, Commission for Education, Science and Technology in Congress, Office of the Vice President of the Republic
B - Foreign Policy	Institution/Organization relevant to Guatemala's foreign policies e.g. Ministry of Foreign Affairs, Central American Parliament...
C - International Partner	Institution/Organization carrying out international cooperation with Guatemala in initiatives relevant to science and technology, e.g. UNESCO, Diplomatic Missions, Cooperation Agencies...
D - Academic Sector (Higher education/Research Centers)	Universities with research activities/research centers, e.g. USAC, UVG, Galileo, Mariano Galvez, URL
E - Industry/Private Sector	Organization/Company, private sector trade association with Research and Development activities, e.g. Cementos Progreso (Cetec, research institute), Agexport (Network I+D+i), Industry Chamber, AGEXPORT, Agribusiness Chamber, CAB-Corpo, CENCIGAÑA, Climate Change Institute ICC and Coffee Research Institute ANACAFE...
F - Civil Society Organizations	Civil Society Organizations e.g. INDESGUA (Instituto para el Desarrollo de la Educación Superior en Guatemala), INCEDES (Instituto Centroamericano de Estudios Sociales y Desarrollo), DEMOS 2025, Heifer International, Plan International, Mercy Corps, WFP, UN Women...

Guiding questions

Part I: Existence/Identification/Characterization/Mapping of the Guatemalan Science Diaspora

Question 1: What ideas, concepts or definitions come to mind with the term "Science Diaspora"?

Question 2: What relationship does your organization/institution have with the topic of the Guatemalan Science Diaspora?

Question 3: Do you personally know of or have you heard of the existence of networks, groups, or identification mechanisms of the Guatemalan Science Diaspora?

- If yes, could you please share the mechanisms, actions or policies you know of?

- If no, why do you think there is this lack?

Question 4: Do you think Artificial Intelligence (AI) could help with the mapping, identification or characterization of the Guatemalan Science Diaspora?

- If yes, how do you think AI can help?

Part II: Relationship of the Guatemalan Science Diaspora with their country of Origin

Question 5: During your career or from your institution/organization, have you collaborated with scientists or experts from Guatemala in the diaspora in any project or in the design and formulation of public policies in Guatemala based on scientific foundations?

Question 6: Do you know of scientific projects or initiatives of other Guatemalans in the Diaspora, or other foreign policy mechanisms (MINEX) or by partner countries, cooperation agencies, or other initiatives in the topics of training or creating connections that are currently being implemented and are related to science or the generation of knowledge in (or for) Guatemala?

Question 7: In your experience, has your organization had any relationship/participation with initiatives to strengthen the National Innovation System (SNI) of Guatemala (e.g. through universities, SENACYT, civil society organizations, foundations, etc) and what role could Guatemalan researchers living abroad play?

Question 8: How can technological/digital platforms connect the scientific diasporas of Guatemala and the different scientific and technological communities of the country? Have you used them? Which would you recommend?

Part III: Policies/Practices/Channels for linking the Guatemalan Science Diaspora with the Development of Guatemala

Question 9: How can knowledge generators (researchers) be connected to users of this knowledge (decision makers, policy makers, broad sectors of the Guatemalan population)? In other words, how can technology best contribute to the production and dissemination of knowledge relevant to the needs of society?

Question 10: Do you know any institution and/or members of networks of the Guatemalan Science Diaspora who work together or collaborate on issues related to public policies or sustainable development projects in Guatemala?

- If yes, what have these mechanisms and/or practices consisted of?

Question 11: Do you know if your institution/organization has been contacted or invited to participate in any group, channel or mechanism to connect the Guatemalan Science Diaspora?

- If yes, what benefits do you consider this type of network brings to the scientific community of the country?

Question 12: What technological tools could be fundamental to create mechanisms, policies and actions to involve the Guatemalan Science Diaspora with the development of Guatemala?

Part IV: Challenges/Barriers/Obstacles to creating Connections

Question 13: What do you consider to be the greatest challenges or barriers to creating connections with the Guatemalan Science Diaspora?

Question 14: What initiatives or actions do you think could reduce the barriers to creating connections with Guatemalan Science Diaspora?

Question 15: Do you know of another study/ies or have you heard of similar research or with similar themes/objectives to the this one?

Question 16: Would you like to mention/recommend another person, institution/organization or network that you think could contribute to this study?

Question 17: Do you have any additional comments/suggestions regarding this study?

Closing: Thank you very much for your participation. Please, do not hesitate to send any additional information at your convenience to the email address owsd.guatemala.chapter@gmail.com

Glossary of key terms: Understanding key terms in the context of this research

Machine learning: discipline in the field of Artificial Intelligence that, through algorithms, equips computers with the ability to identify patterns in big data and make predictions (predictive analysis)

Science Diaspora: Scientists, researchers, engineers, professionals with advanced studies, training and qualifications who reside outside their country of origin or participate in mobility mechanisms for extended periods, particularly in areas of science, technology and education.

Artificial Intelligence: Artificial Intelligence or AI, is a technology that, through a series of algorithms, gives a machine, processor or software the ability to interpret, decide and solve problems autonomously using the input it receives.

Epistemic community: Network or groups of professionals with recognized experience and competence in a particular field. Experts who, due to their specialized knowledge, have legitimacy in the policy area within a given field.

Scientific Advice: It is the process, structures and institutions through which governments, politicians and decision makers consider scientific information and evidence in making decisions and policies. Scientists provide scientific advice.

National Innovation System: Open system made up of organizations, institutions, individuals or communities of individuals in a country that influence the design, development, implementation of policies, strategies, programs, methodologies and mechanisms for the management, promotion, financing, protection and dissemination of scientific research and innovation

T**echnological Platforms*:*** Mechanisms that facilitate the exchange of information, promote participation, and foster cooperation in applied research projects and technology development
